# Characterization of Novel Sigma Receptor Ligands Derived from Multicomponent Reactions as Efficacious Treatments for Neuropathic Pain

**DOI:** 10.3390/ph19010117

**Published:** 2026-01-08

**Authors:** Ryosuke Shinouchi, Bengisu Turgutalp, Rohini S. Ople, Shainnel O. Eans, Ashai K. Williams, Haylee R. Hammond, Andras Varadi, Rebecca Notis Dardashti, Susruta Majumdar, Jay P. McLaughlin

**Affiliations:** 1 Department of Cellular and Systems Pharmacology, College of Pharmacy, University of Florida, Gainesville, FL 32610, USA; r.shinouchi@ufl.edu (R.S.); shaieans@cop.ufl.edu (S.O.E.); williamsashai@ufl.edu (A.K.W.); hhammond@ufl.edu (H.R.H.); 2 Center for Clinical Pharmacology and Department of Anesthesiology, Washington University Pain Center and Washington University School of Medicine, St. Louis, MO 63131, USA; bengisut@wustl.edu (B.T.); rohiniopale@gmail.com (R.S.O.); susrutam@email.wustl.edu (S.M.); 3 Department of Neurology, Molecular Pharmacology and Chemistry, Memorial Sloan Kettering Cancer Center, New York, NY 10065, USArickinotis@gmail.com (R.N.D.)

**Keywords:** sigma, neuropathic pain, inflammatory pain, sigma-1 receptor ligand, allodynia, analgesia, RO-5-3, RO-7-3, multicomponent reactions

## Abstract

**Background/Objectives**: Neuropathic pain remains a significant clinical challenge, with current treatments often providing inadequate relief and adverse effects. Sigma receptors (SRs) modulate nociception and have emerged as potential therapeutic targets for neuropathic pain. Although putative sigma-1 receptor (S1R) ligands have demonstrated analgesic efficacy in preclinical models, their in vivo efficacy and safety profiles require further clarification. **Methods**: Analogs of well-known selective S1R ligand **UVM147** were synthesized using 3-component Ugi reactions and examined in vitro for receptor affinity in radioligand competition binding assays and in vivo with mouse models of neuropathic and inflammatory pain and adverse effects. **Results**: Three novel heterocyclic compounds (**RO-4-3**, **RO-5-3**, and **RO-7-3**) displayed in vitro nanomolar affinity with varying selectivity for both SR subtypes (S1R and S2R). When screened in vivo at a dose of 30 mg/kg s.c. in mice first subjected to chronic constriction injury (CCI), **RO-5-3** and **RO-7-3** possessed anti-allodynic potential, while **UVM147** was inactive. Upon full characterization, **RO-5-3** significantly attenuated mechanical allodynia in a dose-dependent manner, while **RO-7-3** was ineffective at higher doses. Both compounds dose-dependently attenuated nociceptive behaviors in the mouse formalin assay. **RO-5-3** induced mild respiratory depression without impairing locomotor activity, whereas **RO-7-3** caused transient respiratory depression and locomotor impairment. Additionally, **RO-5-3**, but not **RO-7-3**, induced conditioned place aversion consistent with potential S2R involvement. **Conclusions**: **RO-5-3** exerts antinociceptive and anti-allodynic effects with minimal adverse behavioral effects, supporting the role of SRs in pain modulation. These results add to growing evidence supporting the development of SR ligands as efficacious therapeutics for neuropathic pain with fewer clinical liabilities.

## 1. Introduction

Neuropathic pain resulting from damage to somatosensory neurons manifests as allodynia, hyperalgesia, and spontaneous or continuous pain, including paresthesia, dysesthesia, or lightning-like sensations [1,2,3]. It is estimated that neuropathic pain affects approximately 7–10% of the adult population [4]. Neuropathic pain is attributed to a combination of peripheral and central mechanisms, such as those initiated by nerve injury or chemotherapeutic agents [5]. Subsequent peripheral sensitization is known to be driven by increased excitability of primary afferent neurons, for instance, due to altered expression and function of voltage-gated ion channels and transient receptor potential channels [6,7]. In parallel, sustained afferent input to the spinal dorsal horn induces central sensitization, characterized by enhanced excitatory synaptic transmission, diminished inhibitory tone, neuroinflammation, and activation of glial cells [8]. Together, these molecular and cellular processes are believed to contribute to the initiation and persistence of chronic neuropathic pain. Despite its prevalence, treatment options remain limited, and chronic pain management is challenging.

First-line pharmacological treatments for neuropathic pain include tricyclic antidepressants, serotonin-norepinephrine reuptake inhibitors, and gabapentinoids [9,10,11]. Second-line options include lidocaine and high-concentration capsaicin patches, while third-line treatments involve strong opioids [9,10,11]. However, these medications often produce adverse effects, including sedation, dizziness, and motor impairment (first-line agents); erythema and itching (second-line agents); and tolerance, respiratory depression, constipation, dependence, and addiction (with third-line agents)—leading to poor patient compliance [3,12,13]. Moreover, approximately 50% of patients experience inadequate pain relief with current treatments, highlighting the urgent need for novel therapeutics with improved efficacy and safety profiles [11].

Among emerging approaches, sigma receptor (SR) ligands have shown promise as potential therapeutic targets for neuropathic pain [14,15]. Notably, sigma-1 receptor (S1R) inhibitors exert analgesic and anti-allodynic effects, offering a novel strategy for pain treatment [16,17]. Although SRs were once considered part of the opioid family [18], the subsequent cloning of S1R [19] and sigma-2 receptors (S2R) [20] has provided a more precise understanding of their distinct biological nature. S1Rs are thought to modulate pain signaling both centrally and peripherally and function as chaperone proteins in primary sensory neurons [21,22,23]. To date, no approved drug directly targets chaperone activity, making S1R a unique therapeutic target.

S1R expression is elevated in the lumbar dorsal root ganglia under neuropathic conditions, such as spared nerve injury and sciatica [23]. Accumulating evidence suggests that S1R inactivation exerts antinociceptive effects in both inflammatory and neuropathic pain models [24,25,26]. However, many clinically used drugs possessing S1R inhibitory activity, including dextromethorphan, haloperidol, and imipramine, exhibit poor S1R selectivity and significant off-target effects [27]. Recently, the validation of MR309 (E52862) in a phase IIa clinical trial for oxaliplatin-induced neuropathy [28] has renewed interest in developing selective S1R inhibitors as novel therapeutics for neuropathic pain. However, knowledge regarding S2R ligands remains limited. S1R has been extensively validated as a target in neuropathic and inflammatory pain, with selective antagonists such as S1RA/MR309, BD-1047, and BD-1063 showing robust antiallodynic effects in multiple models [29,30], validating the scientific rationale for further study. Building on these findings, our study focuses on evaluating the in vivo pharmacological profiles of **UVM147** and its newly developed analogs with distinct S1R/S2R selectivity.

**Figure 1:**
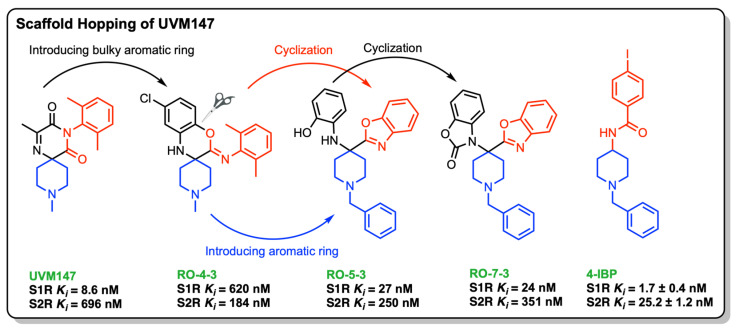
Scaffold hopping strategy of **UVM147** [31] for the design of novel SR ligands (**RO-4-3**, **RO-5-3**, and **RO-7-3**), the established SR ligand 4-IBP [35], their chemical structures, and S1R and S2R binding affinities.

We previously identified a potent S1R ligand, **UVM147**, with an excellent selectivity profile when screened in radioligand competition binding assays against fifty central nervous system (CNS) targets, including G-protein-coupled receptors (GPCRs), ion channels, transporters, and enzymes through the National Institute of Mental Health psychoactive drug screening program (NIMH-PDSP) [31,32]. Possessing a spiro-2,6-dioxopyrazine scaffold synthesized through a three-component Ugi reaction [33,34], **UVM147** offers a new template for the development of potent and selective SR ligands [31]. Through a scaffold hopping approach, we generated three novel SR ligands (**RO-4-3**, **RO-5-3**, and **RO-7-3**) with strong affinity and varying selectivity for S1R and S2R (Figure 1).

The present study aimed to evaluate the in vivo antinociceptive and anti-allodynic effects of potent and selective S1R ligand **UVM147** and its analogs **RO-4-3**, **RO-5-3**, and **RO-7-3**. Preceding behavioral testing, we determined the binding affinities of these compounds for S1R and S2R as well as a number of additional potential targets. In addition, we evaluated potential adverse effects, including respiratory depression, locomotor impairment, and reinforcing properties.

## 2. Results

### 2.1. Generation of Novel S1R Ligands Through Scaffold Hopping

**Scheme 1:**
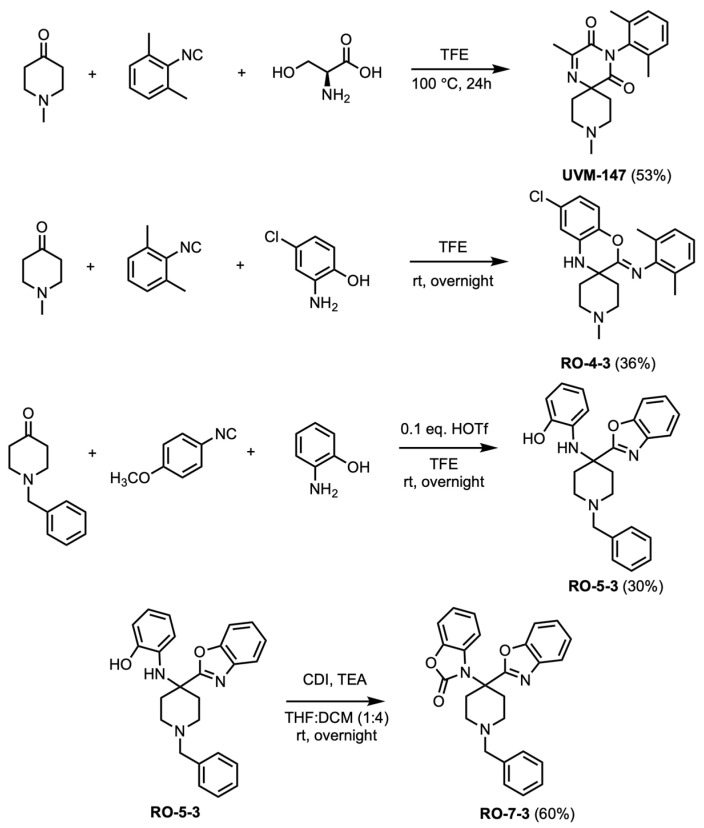
Synthesis of **UVM147** and the three analogs **RO-4-3**, **RO-5-3**, and **RO-7-3**.

The parent compound **UVM147** and analogs **RO-4-3** and **RO-5-3** were synthesized as described previously using a three-component Ugi reaction of a ketone, amine, and isocyanide in slightly acidic protic solvent 2,2,2-trifluoroethanol (TFE) (Scheme 1). The reaction of *N*-methyl-4-piperidone with 2,6-dimethylphenyl isocyanide and β-hydroxy-α-amino acid (serine) under heating conditions results in spontaneous elimination followed by rearrangement, yielding the spiro-2,6-dioxopyrazine scaffold bearing parent compound **UVM147** in 53% yield, as reported previously [31]. To enhance the putative metabolic stability of **UVM147**, we introduced an aromatic ring by using 2-amino-4-chlorophenol as aromatic amine and *N*-methyl-4-piperidone with 2,6-dimethylphenyl isocyanide in 2,2,2-trifluoroethanol solvent at room temperature [33]. An intramolecular nucleophilic attack to the highly reactive nitrilium ion yielded the benzoxazine analog, compound **RO-4-3**, in moderate yield (36%). We then introduced a phenyl ring to the *N*-methyl-4-piperidone moiety to increase the resemblance of our scaffold to well-known S1R antagonist IBP-4 [35] (Figure 1). The benzoxazole analog **RO-5-3** was obtained by reacting *N*-benzyl-4-piperidone with 4-methoxybenzyl isocyanide and excess 2-aminophenol, yielding a nucleophilic attack on the benzoxazine scaffold in more acidic conditions (0.1 equivalent of trifluoromethanesulphonic acid (HOTf), as described previously [33]). We further modified the scaffold by rigidifying the aminophenol moiety of **RO-5-3** to benzoxazolone using carbonyldiimidazole (CDI) in a polar aprotic solvent [33,36] and furnished **RO-7-3** in good yield (60%).

### 2.2. Competition Radioligand Binding and Selectivity for Sigma 1 (S1R) and Sigma 2 (S2R) Receptor

The binding affinities of **UVM147** and the synthesized three analogs for S1R and S2R were determined with competition radioligand binding assays, as was established with the S1R antagonist IBP-4 for comparison (Figure 1). **RO-4-3** displayed significantly reduced S1R binding, yielding S2R selectivity with moderate binding affinity (S2R *K_i_* value = 184 nM and SI (selectivity index) = 3.36). In contrast, compounds **RO-5-3** and **RO-7-3** displayed high affinity for S1R, with *K_i_* values of 27 nM and 24 nM, respectively, comparable to the parent compound **UVM147** (S1R *K_i_* value = 8.6 nM [31]). However, the S1R/S2R selectivity of **RO-5-3** and **RO-7-3** was reduced 10-fold as compared to the parent compound (Figure 1). We then tested the selectivity of **UVM147** and the three analogs against fifty different CNS receptors, including GPCRs, ion channels, transporters, and enzymes, through the screening program (PDSP) offered by the National Institute of Mental Health (NIMH) (Table 1). In contrast with the promiscuity of many sigma ligands, compounds **RO-5-3** and **RO-7-3** displayed excellent S1R selectivity, displaying selectivity index values of at least 15 for the highest binding affinity for CNS off-targets (Table 1).

### 2.3. Alleviation of Mechanical Allodynia in the Mouse Chronic Constriction Injury Model of Neuropathic Pain

**Figure 2:**
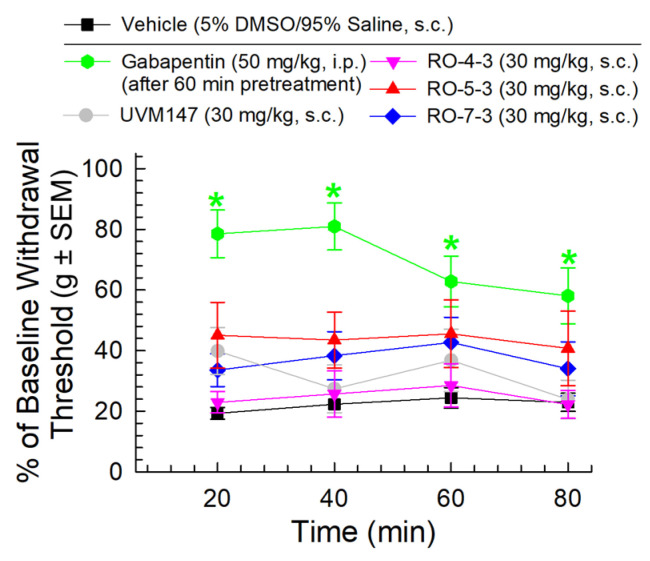
Screening of anti-allodynic effects of the four sigma receptor ligands in the mouse CCI model of neuropathic pain. Mice were administered (at 30 mg/kg, s.c.) **UVM147** (gray circle), **RO-4-3** (pink inverted triangle), **RO-5-3** (red triangle), or **RO-7-3** (blue diamond). Gabapentin (50 mg/kg, i.p., 60 min pre-treatment; green hexagon) was included as a positive control; vehicle (5% DMSO/95% saline, s.c.; black square) was included as a negative control. Withdrawal response expressed as a percentage of initial baseline (non-allodynic) withdrawal thresholds measured using the von Frey test. Data represent the mean ± SEM (*n* = 8–13 for all groups/points). * = significantly different from vehicle controls at matching time points, *p* < 0.05; two-way ANOVA with Tukey’s post hoc test.

The four synthesized sigma receptor ligands **UVM147**, **RO-4-3**, **RO-5-3**, and **RO-7-3** were screened at a 30 mg/kg, s.c., dose in the mouse chronic constriction injury (CCI) model of neuropathic pain (Figure 2). Following surgery and confirmation of induced mechanical allodynia, one of each of the four SR ligands was administered, and mechanical allodynia was assessed every 20 min for 80 min using von Frey filaments. The established treatment for neuropathic pain, gabapentin was administered as a positive control (50 mg/kg, i.p., given 60 min prior to testing). Treatment significantly attenuated the reduced paw withdrawal threshold caused by CCI (*n* = 8–13 per group; factor: treatment: F_(5, 55)_ = 9.316, *p* < 0.0001; factor: time: F_(2.695, 148.2)_ = 2.347, *p* = 0.08; RM two-way ANOVA with Tukey’s multiple comparison post hoc test; Figure 2). Notably, although **RO-5-3** and **RO-7-3** in general attenuated the reduced withdrawal threshold, none of the SR ligands significantly attenuated the reduced paw withdrawal threshold at any specific time point (*p* > 0.05, n.s., vs. matching vehicle response; Tukey’s multiple comparison post hoc test).

**Figure 3:**
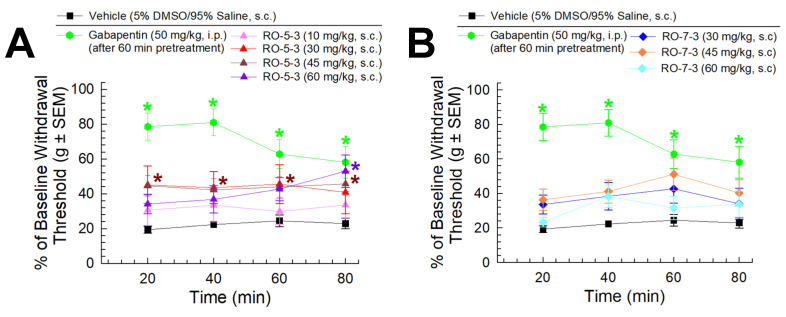
Dose- and time-dependent anti-allodynic effects in the mouse CCI assay of (**A**) **RO-5-3** (10, 30, 45, and 60 mg/kg, s.c.; triangles) or (**B**) **RO-7-3** (30, 45, and 60 mg/kg, s.c.; diamonds). Gabapentin (50 mg/kg, i.p., 60 min pre-treatment; green hexagon) was included as a positive control; vehicle (5% DMSO/95% saline, s.c.; black square) was included as a negative control. Withdrawal response expressed as a percentage of initial baseline (non-allodynic) withdrawal thresholds measured using the von Frey test. The data are represented as the mean ± SEM (*n* = 9–13 for all groups/points). * = significantly different from vehicle controls at matching time points, *p* < 0.05; two-way ANOVA with Dunnett’s post hoc test.

Further dose-dependent effects of the two most active SR ligands, **RO-5-3** and **RO-7-3**, were examined in the mouse CCI assay. Following administration, **RO-5-3** (Figure 3A) demonstrated significant anti-allodynic effects in the CCI model after treatment with higher doses (45 and 60 mg/kg, s.c.; *n* = 9–13 per group; treatment × time: F_(15, 180)_ = 2.12, *p* = 0.01; RM two-way ANOVA with Dunnett’s multiple comparison post hoc test), although this seemed to reach a ceiling effect at the 45 mg/kg, s.c., dose (*p* < 0.05 vs. vehicle response at matching time point; Dunnett’s post hoc test). Likewise, **RO-7-3** (Figure 3B) demonstrated significant anti-allodynic effects in the CCI model after treatment (*n* = 9–13 per group; factors: treatment × time: F_(12, 147)_ = 2.02, *p* = 0.03; RM two-way ANOVA), although these effects were not significantly different at any specific time point compared to vehicle (*p* > 0.05, n.s.; Dunnett’s multiple comparison post hoc test).

### 2.4. RO-5-3 and RO-7-3 Induce Dose-Dependent Antinociception in the Mouse Formalin Model of Inflammatory Nociception

**Figure 4:**
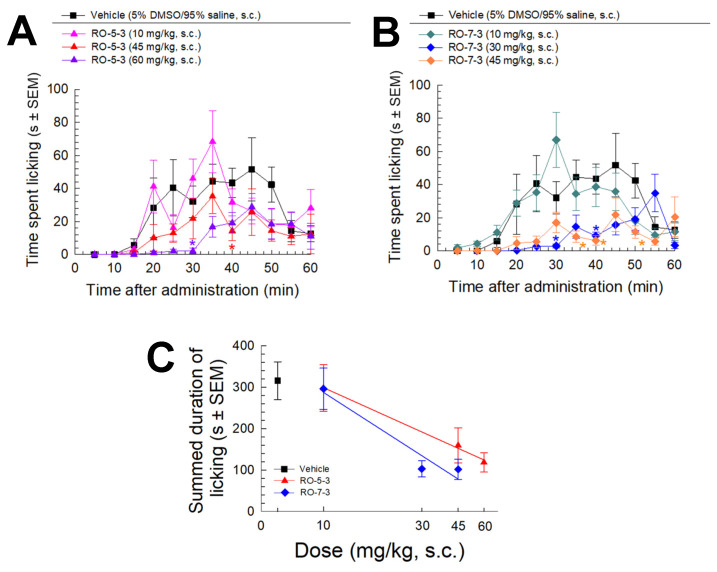
Time- and dose-dependent antinociception of **RO-5-3** and **RO-7-3** following s.c. administration in the mouse formalin assay. Summed duration of licking in 5 min bins over 60 min was assessed after administration of (**A**) **RO-5-3** (10, 45, and 60 mg/kg, s.c.; triangles) and (**B**) **RO-7-3** (10, 30, and 45 mg/kg, s.c.; diamonds) in C57BL/6J mice, with (**C**) average responses by dose. Vehicle (5% DMSO/95% saline, s.c.; black square) is included as a negative control. The data are represented as the mean ± SEM (*n* = 10 for all groups/points). * = significantly different from vehicle controls at matching time points, *p* < 0.05; two-way ANOVA with Dunnett’s post hoc test.

Based on their anti-allodynic activity in the neuropathic pain model, we examined the antinociceptive efficacy of **RO-5-3** (10, 45, and 60 mg/kg, s.c.) and **RO-7-3** (10, 30, and 45 mg/kg, s.c.) in the mouse formalin assay of inflammatory pain (Figure 4). Treatment with **RO-5-3** (Figure 4A) and **RO-7-3** (Figure 4B) significantly reduced the amount of time animals spent licking the inflamed paw in a dose-dependent manner (*n* = 10 per group; **RO-5-3**; F_(3, 36)_ = 5.124, *p* = 0.005; **RO-7-3**; F_(3, 36)_ = 10.13; *p* < 0.0001; one-way ANOVA with Dunnett’s post hoc test over time compared to vehicle-treated mice, with an ED_50_ (and 95% C.I.) value of 35.7 (20.0–83.0) mg/kg, s.c., and 20.7 (16.1–25.7) mg/kg, s.c., respectively (Figure 4C)).

### 2.5. Evaluation of RO-5-3 and RO-7-3 for Potential Respiratory and Locomotor Impairment

**Figure 5:**
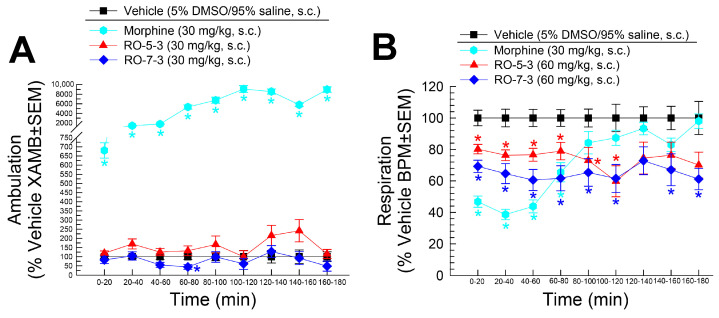
Evaluation of (**A**) spontaneous ambulation and (**B**) respiration in C57BL/6J mice administered sigma receptor ligands **RO-5-3** or **RO-7-3**. Sigma receptor ligands were tested for ambulatory effects (**A**) at 30 mg/kg and effects on breathing rate (**B**) using the CLAMS assay. Morphine (30 mg/kg, s.c.; cyan hexagon) is included as a positive control; vehicle (5% DMSO/95% Saile, s.c.; black square) is included as a negative control. Data are represented as the mean ± SEM (*n* = 12–16 for all groups/points). * = significantly different from vehicle controls at matching time points, *p* < 0.05; two-way ANOVA with Dunnett’s post hoc test.

Using the Comprehensive Lab Animal Monitoring System (CLAMS), we evaluated spontaneous locomotor activity and respiratory effects. Psychostimulant and positive control morphine (30 mg/kg, s.c.) significantly increased ambulation from 20 to 180 min post-treatment (*n* = 12–16 per group; treatment × time: F_(24, 448)_ = 142.30; *p* < 0.0001; two-way RM ANOVA with Dunnett’s multiple comparisons post hoc test; Figure 5A). In contrast, **RO-5-3** (30 mg/kg, s.c.) produced no significant changes in spontaneous ambulation at any time point, while **RO-7-3** (30 mg/kg, s.c.) produced a small, if statistically significant, increase in ambulation at the 60–80 min time point. Characteristic of mu opioid agonists, morphine also significantly reduced respiration rates between 0–80 min post-injection (*n* = 12–16 per group; treatment: F_(24, 448)_ = 6.96; *p* < 0.0001; two-way RM ANOVA with Dunnett’s multiple comparisons post hoc test; Figure 5B). Notably, although high doses of **RO-5-3** and **RO-7-3** (60 mg/kg, s.c.) significantly reduced respiration rates compared to vehicle-treated mice longer (120–140 min) than morphine, the extent of respiratory depression induced by either compound was significantly less than that observed with morphine during the initial 80 min (Figure 5B). Even at half the dose, morphine induced significantly greater reductions in breathing rate over ***RO-5-3*** (F_(8, 208)_ = 17.64, *p* < 0.0001; two-way ANOVA) for the first hour (*p* < 0.0001; Sidak’s post hoc test; Figure 5B) but no other time point.

**Figure 6:**
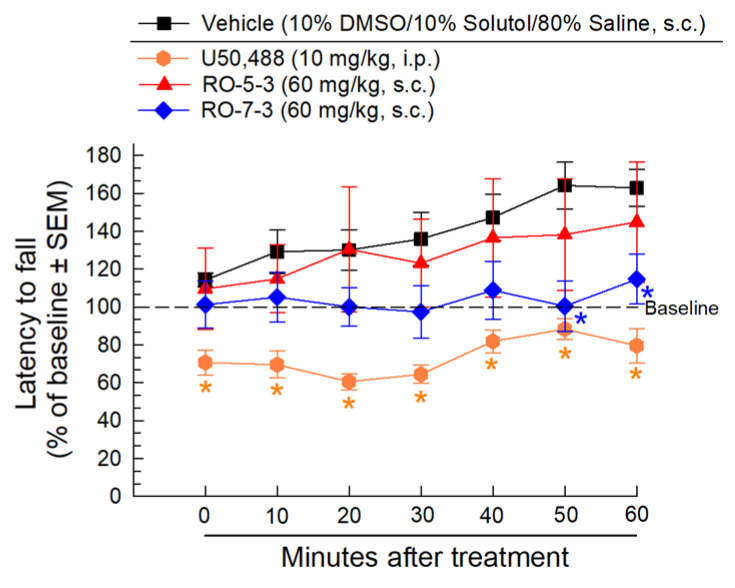
Evaluation of the **RO-5-3** (60 mg/kg, s.c.; red triangle) and **RO-7-3** (60 mg/kg, s.c.; blue diamond) after administration in the C57BL/6J mouse rotarod assay. U50,488 (10 mg/kg, i.p.; orange hexagon) is included as a positive control; vehicle (10% DMSO/10% Solutol/80% saline, s.c.; black square) is included as a negative control. The data are represented as the mean ± SEM (*n* = 8–10 for all groups/points). * = significantly different from vehicle controls at matching time points, *p* < 0.05; two-way ANOVA with Dunnett’s post hoc test.

Rotarod testing was used to evaluate sedative-like or locomotor-impairing effects. The kappa opioid receptor (KOR)-selective agonist U50,488 served as a positive control, significantly impairing evoked locomotion compared to mice treated with vehicle (*n* = 8–10 per group; treatment: F_(3, 30)_ = 4.19, *p* < 0.01; time: F_(4.874, 146.2)_ = 8.45, *p* < 0.0001; two-way RM ANOVA with Dunnett’s multiple comparisons post hoc test; Figure 6). **RO-5-3** (60 mg/kg, s.c.) did not significantly impair locomotion or produce sedative-like effects at any time point, although **RO-7-3** (60 mg/kg, s.c.) significantly impaired locomotion at the 50- and 60 min time points compared to vehicle-treated mice (*p* < 0.05; Dunnett’s post hoc test).

### 2.6. Evaluation of RO-5-3 and RO-7-3 in the Mouse Conditioned Place Preference Assay

**Figure 7:**
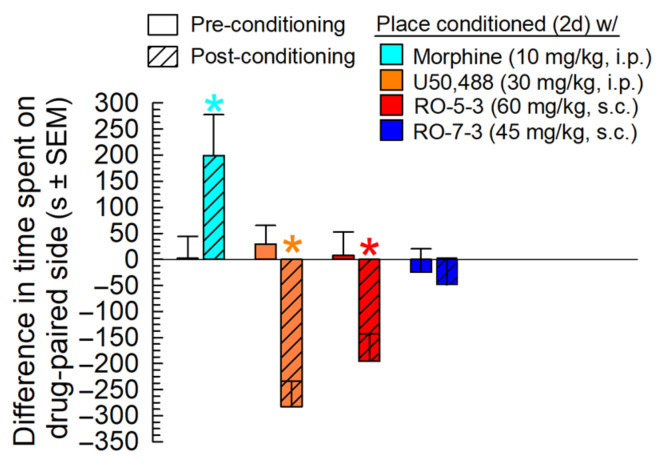
Evaluation of the sigma receptor ligands **RO-5-3** (60 mg/kg, s.c.; red) and **RO-7-3** (45 mg/kg, s.c.; blue) in the conditioned place preference (CPP) assay. Control C57BL/6J mice received place conditioning with morphine (10 mg/kg, i.p.; cyan) or the aversive KOR agonist, U50,488 (30 mg/kg, i.p.; orange). Data represent mean ± SEM (*n* = 18–28 for all groups). * = post-conditioning response (striped bars) significantly different from matching pre-conditioning response (matching open bars), *p* < 0.05; two-way ANOVA with Sidak’s post hoc test.

Mice were place conditioned for 40 min on each of two days with morphine (10 mg/kg, i.p.), the KOR-selective agonist U50,488 (30 mg/kg, i.p.), or the sigma receptor ligands **RO-5-3** (60 mg/kg, s.c.) or **RO-7-3** (45 mg/kg, s.c.). Morphine produced significant conditioned place preference (CPP), and U50,488 produced conditioned place aversion (CPA) (*n* = 18–28 per group; factor: treatment × conditioning: F_(3, 90)_ = 11.4; *p* < 0.0001; two-way ANOVA with Sidak’s multiple comparisons post hoc test). Whereas **RO-5-3** produced significant CPA (*p* = 0.007; Figure 7), **RO-7-3** produced place-conditioning responses that did not change from pre-conditioning responses (*p* = 0.99; Figure 7).

## 3. Discussion

In this study, we synthesized and characterized three novel analogs of our previously reported [31] potent and selective S1R ligand **UVM147** (S1R *K_i_* = 8.6 nM with SI = 81). Introducing a chlorine-substituted aromatic ring resulted in reduced S1R binding affinity but preserved S2R affinity, yielding **RO-4-3** as an S2R-selective ligand (S2R *K_i_* = 184 nM, SI = 3). This could result from reduced stability of the benzoxazine-2-imine scaffold being prone to nucleophilic attack. Increasing the resemblance of our scaffold to 4-IBP through incorporation of an aromatic ring to the piperidine moiety restored S1R binding affinity. Compounds **RO-5-3** and **RO-7-3** displayed potent S1R affinity but reduced selectivity compared to UVM147 (S1R *K_i_* = 27 nM with SI = 9, and S1R *K_i_* = 24 nM with SI = 15, respectively). This suggests the introduction of an additional aromatic ring to the scaffold plays a key role in S1R selectivity (Figure 1, highlighted as black). We also performed off-target profiling for the compounds against the main CNS drug target receptors. Similar to the parent compound UVM147 [31], compounds demonstrated a good selectivity profile (Table 1).

We then investigated the anti-allodynic properties of our compounds **UVM147**, **RO-4-3**, **RO-5-3**, and **RO-7-3**. Among them, **RO-5-3** demonstrated both anti-allodynic and antinociceptive effects but also mildly depressed respiration and produced conditioned place aversion, although it did not affect locomotor activity or motor coordination.

When screened in vivo at a 30 mg/kg, s.c., dose in CD-1 mice subjected to chronic constriction injury (CCI) of the sciatic nerve, **RO-5-3** and **RO-7-3** possessed anti-allodynic potential. Upon full characterization, higher doses (45 and 60 mg/kg) of **RO-5-3**, but not **RO-7-3**, significantly attenuated mechanical allodynia in the CCI model of neuropathic pain. The CCI model is a commonly used rodent model of neuropathic pain resulting from sciatica [37,38,39]. Gabapentin, established as a “gold standard” treatment for neuropathic pain [40,41,42], exhibited anti-allodynic effects at a dose of 50 mg/kg i.p. following a one-hour pre-treatment to avoid confounding sedative effects, consistent with previous CCI studies [40]. **RO-5-3**, which exhibits high binding affinity and moderate selectivity for S1R, showed anti-allodynic effects in the CCI model at doses of 45 and 60 mg/kg, albeit less potent than gabapentin, similar to previously reported data with SR antagonists [40,43,44]. A full dose–response assessment was not performed because the study design focused on initial evaluation of efficacy at doses selected based on in vitro affinity, compound solubility, and prior pharmacological knowledge. Quantitative parameters such as ED_50_ could not be calculated from the limited dose–response range available, but future studies employing multiple dose levels of salt versions of the compounds would be expected to more fully enable comparative efficacy. CCI, which involves localized injury to the sciatic nerve, has been shown to up-regulate S1R expression in the spinal cord, promote central sensitization, and activate microglia within the spinal cord and supraspinal regions of the brain [45,46]. Additionally, it has been shown to increase mechanical allodynia in parallel with an increase in S1R immunostained GFAP-positive cells in the superficial dorsal horn (laminae I–II) region of the spinal cord, which is reduced by S1R inhibition [47]. These results suggest the anti-allodynic effects of **RO-5-3** may be mediated by a down-regulation of SR expression in the spinal cord, although more testing is required to examine this.

**RO-5-3** and **RO-7-3**, consistent with previous studies utilizing S1R ligands, attenuated acute non-reflexive inflammatory pain in the formalin assay [40,44,48]. The initial short-lived acute pain response associated with formalin administration (Phase I, considered to result from immediate stimulation of peripheral C-fibers) [49] is followed by Phase II, characterized by a prolonged period of persistent nociception [50,51], which is associated with neuronal sensitization in the spinal cord due to afferent nociceptor stimulation [52]. Inhibition of Phase II formalin-induced pain by **RO-5-3** and **RO-7-3** is consistent with reports indicating the involvement of S1R in sensitization to nociception [53,54]. Collectively, these recent data suggest that the functions of **RO-5-3** and **RO-7-3** align with the antinociceptive properties demonstrated by other S1R ligands [40,43,44,55].

Currently, gabapentin and morphine are utilized for the treatment of neuropathic pain, but both produce significant liabilities as well, including reduced respiratory rate and locomotor activity, as well as the potential for dependence and mortality in some cases [56,57,58]. In this study, we evaluated the potential adverse effects of **RO-5-3** and **RO-7-3** using respiratory depression, rotarod, and conditioned place preference/aversion (CPP/CPA) assays. Using the CLAMS, we measured breathing rate and spontaneous locomotor activity. Morphine exhibited the expected spontaneous hyperlocomotion and severe respiratory depression [59,60]. In contrast, **RO-5-3** did not exhibit the adverse effect of spontaneous hyperlocomotion but did induce mild reductions in breathing rate, albeit less severe than that induced by morphine. **RO-7-3** also modestly depressed breathing and caused transient reductions in spontaneous locomotor activity compared to **RO-5-3**. These results are consistent with previous reports of dual S1R/S2R antagonism by AZ-66 [40]. Moreover, these effects have been suggested to arise from the S1R dimerization with and activation of opioid receptors located in the brainstem, where S1R is highly concentrated [61], and the medulla and hypothalamus, which mediate arousal and sedation, suggesting possible sigma receptor modulation in these behaviors.

To confirm the absence of locomotor impairment, we further tested the ability of **RO-5-3** and **RO-7-3** to impair induced locomotor activity using the rotarod assay. U50,488 exhibited motor incoordination and sedation [62,63]. **RO-5-3**, similar to the S1R antagonist CM-304, did not impair induced locomotor activity [40]. However, **RO-7-3** transiently impaired induced locomotor activity compared to the vehicle group. Considering these results, consistent outcomes regarding compound-induced locomotor impairment have not been obtained when evaluating various S1R ligands at high doses [40,44]. Thus, the potential mechanisms by which S1R ligands affect induced locomotor activity remain unclear. As more S1R-selective ligands are developed and become available for evaluation, it will be possible to comprehensively predict whether S1R/S2R-selective ligands exhibit sedative effects.

Given the high abuse potential of opioids, cocaine, and psychostimulants [64], drug abuse and dependence remain critical concerns in the use of analgesics [65]. To assess the potential rewarding or aversive effects of **RO-5-3** and **RO-7-3**, we utilized the CPP/CPA assay. Consistent with previous reports, morphine exhibited typical rewarding effects [66,67], whereas the KOR agonist U50,488 induced typical aversive effects [68]. **RO-7-3** did not induce aversion, although **RO-5-3** induced CPA similar to U50,488. Interestingly, **RO-5-3**, but not **RO-7-3**, induced CPA at the highest dose. Notably, no signs of sedation or malaise were observed, suggesting that this aversion is unlikely to reflect nonspecific behavioral suppression, and **RO-5-3** displayed less affinity for non-sigma targets than **RO-7-3**. One potential explanation is differential S2R engagement, as structurally related analogs have been reported to vary markedly in S2R affinity despite comparable S1R profiles [43]. Further testing, such as in transgenic mice lacking S1R or S2R, would be expected to help resolve this question and remains a priority for future testing. Notably, this finding with **RO-5-3** was inconsistent with previous reports indicating that S1R ligands do not induce reward or aversion [40,44] but is consistent with the reported action of the S2R-selective antagonist CM398 [43]. These data suggest that compounds with affinity for S2R may exhibit aversive effects. While beyond the scope of the current study, behavioral testing using S1R and S2R knockout mice is expected to provide new insights by clarifying the specific contributions of S1R and S2R to reward or aversive states. Although these initial studies demonstrate useful interactions, a limitation of the present results is that receptor-specific mediation of the antiallodynic and antinociceptive effects could not be directly confirmed. Future experiments using S1R-knockout animals or selective S1R/S2R pharmacological tools will be of value to definitively determine the receptor subtype responsible for the observed effects and their respective contributions.

In addition to observed behavioral results, future experiments examining the pharmacokinetic parameters, including plasma/brain exposure, half-life, and free drug concentrations, will be extremely valuable moving forward. Establishing the pharmacokinetic/pharmacodynamic relationship, including brain penetration and target engagement, will uncover some of the mechanisms by which **RO-5-3** and **RO-7-3** exert their effects. Given that **RO-5-3** and **RO-7-3** are newly synthesized compounds, pharmacokinetic parameters are not currently available. However, both compounds exhibit behaviorally active concentrations under the experimental conditions of this study, providing proof-of-concept for differences in S1R/S2R selectivity.

The ongoing opioid crisis continues to drive significant demand for the development of non-opioid therapeutics for pain management [69,70]. Preclinical and clinical studies employing S1R ligands suggest their potential as promising treatments for chronic neuropathic pain [16,17,24,25,26,28,71].

As S1R is a chaperone receptor, it is not constrained by established pharmacological mechanisms governing receptor function, leading to ongoing questions regarding the precise mechanisms of action of S1R ligands. To this point, the unusual pharmacology of sigma receptors has hindered the development of conventional in vitro high-throughput screening assays, making the classification of SR ligands as agonists/antagonists difficult. Currently, there is a lack of reliable in vitro methods for determining S1R functionality; therefore, the present study has relied on validated methods of in vivo characterization. Historically, the antinociceptive effects of S1R ligands have been attributed to the ability of S1R to interact with NMDA [72] receptors, opioid [18] receptors, and ion channels such as calcium and potassium channels [73], but each of these findings poses limitations as yet not resolved [27]. Interactions with other proteins may explain some of the antagonistic actions observed in inflammation assays while potentially diminishing anti-neuropathic effects. Although elucidating the affinities of these compounds for other receptor and ion channel targets associated with antinociceptive actions falls outside the scope of the current study, it remains a priority for future research.

Currently, these data support the development of S1R ligands with modest selectivity as therapeutics for the treatment of chronic pain. Supporting this, S1R is known to be highly expressed in major regions of the central and peripheral nervous systems, particularly in the dorsal root ganglia, where it plays a critical role in modulating the transmission, conduction, and perception of pain signals [27,74]. While the precise mechanisms by which S1R modulates neuropathic and acute inflammatory nociception remain unclear, it is noteworthy that S1R ligands have been shown to have minimal effects on altering normal pain sensitivity thresholds, aligning with the reduced side effects, such as sedation, observed with these agents.

## 4. Materials and Methods

### 4.1. Chemistry

All reagents, starting materials, and solvents were purchased from Sigma Aldrich Chemicals (St. Louis, MO, USA), Ambeed (Buffalo Grove, IL, USA), Chemscene (Monmouth Junction, NJ, USA), or Chemimpex (Wood Dale, IL, USA) and used as such without further purification. Air-sensitive reagents and solutions were transferred to glass apparatus using syringe or cannula via rubber septa. The progress of reactions was monitored using thin-layer chromatography (TLC) with 0.25 mm precoated silica gel plates (60 F254). TLCs were visualized with UV light. Reaction mixtures were purified by silica flash chromatography on E. Merck 230−400 mesh silica gel 60 using a Teledyne ISCO Combi Flash Rf instrument (Teledyne ISCO, Lincoln, NE, USA) with UV detection at 280 and 254 nm using 0–10% methanol in DCM solvent system. RediSep Rf silica gel normal-phase columns (Teledyne ISCO, Lincoln, NE, USA) were used. The yields reported are isolated yields. All ^1^H NMR and ^13^C NMR spectra were recorded using a Varian 400 MHz spectrometer (Varian Inc., Palo Alto, CA, USA) at Washington University School of Medicine in St. Louis collected via the Bruker Topspin Software (Bruker Topspin 3.5 pI 6). Coupling constants (J) were measured in Hertz (Hz). NMR spectra were processed with Mestre Nova software (ver. 10.0.2.). Chemical shifts are reported in parts per million (ppm) relative to residual solvent peaks rounded to the nearest 0.01 for proton and 0.1 for carbon (CDCl_3_ ^1^H: 7.26, ^13^C: 77.16). The following abbreviations were used to explain multiplicities: s = singlet, d = doublet, t = triplet, q = quartet, m = multiplet, and br = broad. Mass spectra were obtained at the St. Louis College of Pharmacy using the Agilent 1100 Series LC/MSD (Agilent Technologies, Santa Clara, CA, USA) by electrospray (ESI) ionization with a gradient elution program (Ascentis Express Peptide C18 column (Supelco Inc, Bellefonte, PA, USA) acetonitrile/water 5/95/95/5, 0.05% formic acid, 5 min) and UV detection (214 nM/254 nM). Accurate masses are reported for the molecular ion [M+H]^+^. High-resolution mass spectra were recorded using positive-ion mode electrospray ionization with an Apollo II ion source on a Bruker 10 Tesla APEX-Q exactive FTICR-MS (Bruker, Billerica, MA, USA). HPLC data were generated by Agilent 1100 HPLC (Agilent Technologies, Santa Clara, CA, USA), using a Waters Sunfire C18 3.5 μm pore-size, 4.6 × 50 mm column (Waters Corp, Milford, MA, USA). LC chromatograms were processed with Mestre Nova software (ver. 14.3.3) The analysis was performed using a linear gradient of 5–95% acetonitrile containing 0.05% TFA (Solvent B) against 0.05% trifluoroacetic acid in water (Solvent A) over 10, or 20 min, followed by an isocratic hold at 95% B for 1 min. The flow rate was maintained at 1.0 mL/min, and the injection volume was 5 µL. Peak detection was monitored by 210 and 254 nm UV-absorption. Chemical nomenclature was generated using Chem Bio Draw Ultra 13.0.

#### 4.1.1. Synthesis of 4-(2,6-Dimethylphenyl)-2,9-dimethyl-1,4,9-triazaspiro[5.5]undec-1-ene-3,5-dione (UVM147 CAS No: 2262446-43-3)

**UVM147** was prepared as reported [31]. Briefly: *N*-Methyl-4-piperidone (215 mg, 1.9 mmol) was dissolved in trifluoroethanol (4.0 mL) at room temperature. Serine (200 mg, 1.9 mmol) and 2,6-dimethylphenyl isocyanide (249 mg, 1.9 mmol) were added to the solution. Triethylamine (0.26 mL, 1.9 mmol) was charged into the reaction mass, and the temperature of the reaction was raised to and maintained at 100 °C. The progress of the reaction was monitored under TLC. After 24 h, the reaction mixture was cooled to room temperature, and the solvent was removed under reduced pressure. Then, the mixture was redissolved in dichloro-methane (DCM) (0.5 mL) and was directly purified by using silica gel column chromatography with 0–10% MeOH in DCM. The product fraction, based on mass spectrometry, was collected, solvent was removed, and the content was dried under reduced pressure to obtain **UVM-147** (316 mg, 53%). Product was obtained as a yellow solid (316 mg, 53% yield). ^1^H NMR (400 MHz, CDCl_3_) δ ppm 7.25–7.19 (m, 1H), 7.13 (d, *J* = 7.6 Hz, 2H), 2.87–2.69 (m, 4H), 2.57–2.46 (m, 2H), 2.43 (s, 6H), 2.00 (s, 6H), 1.78 (d, *J* = 12.9 Hz, 2H). ^13^C NMR (100 MHz, CDCl_3_) δ ppm 175.18, 174.49, 157.02, 155.51, 135.09, 131.7, 129.35, 128.75, 63.53, 49.96, 46.17, 36.76, 21.73, 17.83.

#### 4.1.2. Synthesis of (E/Z)-6-Chloro-N-(2,6-dimethylphenyl)-1′-methyl-2H,4H-spiro[benzo[b][1,4]oxazine-3,4′-piperidin]-2-imine (RO-4-3)

**RO-4-3** was prepared according to the modified reported procedure [33] as follows: *N*-Methyl-4-piperidone (200 mg, 1.76 mmol) was dissolved in trifluoroethanol (10 mL) at room temperature. To the solution, 2-amino-4-chlorophenol (252 mg, 1.76 mmol) and 2,6-dimethylphenyl isocyanide (230 mg, 1.76 mmol) were added. The reaction mixture was stirred at room temperature overnight. The next day, the solvent was evaporated under reduced pressure. The mixture was then redissolved in DCM (0.5 mL) and was directly purified by using silica gel column chromatography with 0–10% MeOH in DCM. The product fraction, based on mass spectrometry, was collected, solvent was evaporated, and the content was dried under reduced pressure to obtain **RO-4-3** (237 mg, 36%). Product was obtained as a brown solid (237 mg, 36% yield). ^1^H NMR (400 MHz, CDCl_3_) δ ppm 7.02 (d, *J* = 7.4 Hz, 2H), 6.91 (t, *J* = 7.5 Hz, 1H), 6.77 (s, 1H), 6.69 (s, 2H), 4.14 (s, 1H), 2.83 (d, *J* = 10.2 Hz, 2H), 2.72 (t, *J* = 6.0 Hz, 1H), 2.47 (t, *J* = 6.0 Hz, 1H), 2.42–2.36 (m, 4H), 2.36 (s, 3H), 2.08 (s, 6H), 1.86 (d, *J* = 12.0 Hz, 2H). ^13^C NMR (100 MHz, CDCl_3_) δ ppm 174.51, 151.73, 143.77, 140.23, 132.31, 128.58, 127.70, 127.52, 123.14, 119.79, 116.96, 115.52, 55.48, 51.84, 51.21, 46.28, 45.61, 41.28, 33.04, 18.42. HRMS (ESI) *m*/*z* calculated for [C_21_H_24_ClN_3_O]^+^ ([M+H]^+^): 370.168067; found: 370.167943 (see Supplemental Materials).

#### 4.1.3. Synthesis of 2-((4-(Benzo[d]oxazol-2-yl)-1-benzylpiperidin-4-yl)amino)phenol (RO-5-3)

**RO-5-3** was prepared according to the modified reported procedure [33] as follows: *N*-Benzyl-4-piperidone (0.85 mL, 4.59 mmol) was dissolved in trifluoroethanol (20 mL) at room temperature. To the solution, 2-aminophenol (1.50 g, 13.76 mmol) and 2,6-dimethylphenyl isocyanide (610 mg, 4.59 mmol) were added. Triflic acid (40 µL, 0.459 mmol) was added to the reaction mixture dropwise. The reaction mixture was stirred at room temperature overnight. The next day, the solvent was removed under reduced pressure. The mixture was then redissolved in DCM (20 mL) and washed with saturated NaHCO_3_ solution and then brine (3 × 20 mL). The organic layer was dried with sodium sulfate, concentrated under reduced pressure, and purified by using silica gel column chromatography with 0–10% MeOH in DCM. The product fraction, based on mass spectrometry, was collected, solvent was removed, and the content was dried under reduced pressure to obtain **RO-5-3** (550 mg, 30%). Product was obtained as a pale-yellow solid (550 mg, 30% yield). ^1^H NMR (400 MHz, CDCl_3_) δ ppm 7.77–7.73 (m, 1H), 7.52–7.49 (m, 1H), 7.36–7.28 (m, 7H), 6.79 (s, 2H), 6.62–6.58 (m, 1H), 6.50 (d, *J* = 7.8 Hz, 1H), 3.59 (s, 2H), 2.80 (bs, 2H), 2.59 (bs, 4H), 2.19 (bs, 2H). ^13^C NMR (100 MHz, CDCl_3_) δ ppm 174.52, 150.85, 149.36, 143, 140.5, 132.15, 129.55, 128.55, 127.6, 125.42, 124.69, 123.03, 121.58, 120.57, 120.17, 115.96, 111.07, 62.95, 56.47, 49.43, 34.3. HRMS (ESI) calculated for [C_25_H_25_N_3_O_2_]^+^ ([M+H]^+^): 400.201954; found: 400.201827 (see Supplemental Materials).

#### 4.1.4. Synthesis of 3-(4-(Benzo[d]oxazol-2-yl)-1-benzylpiperidin-4-yl)benzo[d]oxazol-2(3H)-one (RO-7-3)

**RO-7-3** was prepared according to the modified reported procedures [33,36] as follows: **RO-5-3** (500 mg, 1.25 mmol) was dissolved in anhydrous tetrahydrofuran–DCM (1:4, 25 mL). To the solution, carbonyldiimidazole (0.220 mg, 1.376 mmol) was added, and triethylamine (0.35 mL, 2.5 mmol) added dropwise. The reaction was stirred overnight at room temperature. The next day, reaction solvent was evaporated under reduced pressure. Residue was dissolved in DCM (20 mL) and washed with brine (3 × 20 mL). The organic layer was dried with sodium sulfate, concentrated under reduced pressure, and purified by using silica gel column chromatography with 0–10% MeOH in DCM. The product fraction, based on mass spectrometry, was collected, solvent was evaporated, and the content was dried under reduced pressure to obtain **RO-7-3** (320 mg, 60%). Product was obtained as a pale-yellow solid (320 mg, 60% yield). ^1^H NMR (400 MHz, CDCl_3_) δ ppm: 7.81–7.79 (m, 1H), 7.50–7.48 (m, 1H), 7.37–7.30 (m, 6H), 7.27 (bs, 1H), 7.17 (d, *J* = 7.79 Hz, 1H), 7.05 (t, *J* = 7.4 Hz, 2H), 6.98 (t, *J* = 8 Hz, 1H), 3.51 (s, 2H), 3.12 (t, *J* = 9.2 Hz, 2H), 2.96 (bs, 2H), 2.77 (bs, 2H), 2.60 (t, *J* = 8.78 Hz, 2H). ^13^C NMR (100 MHz, CDCl_3_) δ ppm: 174.44, 164.29, 152.80, 150.71, 142.66, 140.40, 138.35, 130.28, 129.07, 128.37, 127.22, 125.88, 124.78, 123.62, 122.70, 120.65, 111.88, 111.15, 110.08, 62.75, 60.92, 50.11, 32.94. HRMS-(ESI) m/z, calculated for [C_26_H_24_N_3_O_3_]^+^ ([M+H]^+^): 426.181218; found: 426.181097 (see Supplemental Materials).

### 4.2. Evaluation of Compound Affinity with Radioligand Competitive Binding Assays

The binding affinities of **UVM147** and the three analogs were determined by radioligand competition binding assays conducted by the National Institute of Mental Health Screening Program (NIMH-PDSP) [31,32]. Details of the methods used for the binding assays are available on the NIMH-PDSP website at https://pdspdb.unc.edu/pdspweb/content/PDSP%20Protocols%20II%202013-03-28.pdf (accessed on 25 October 2025).

### 4.3. Animal Subjects

Male C57BL/6J (The Jackson Laboratory, Bar Harbor, ME, USA) and CD-1 (Charles River Laboratories, Wilmington, MA, USA) mice aged 8–12 weeks were housed five per cage and used in these studies. C57BL/6J mice were used in the locomotor and respiration assays [40,75], rotarod assay [43,44,76], formalin assay [40,43,44,48,49,50,51,76], and conditioned place preference assay [40,43,44,77,78]. CD-1 mice were used to verify the antiallodynic effects in the chronic constriction nerve injury (CCI) assay of neuropathic pain [40,79,80,81].

All animal studies were executed and reported in accordance with the ARRIVE guidelines [82]. Animals were randomly assigned to treatment groups in a blinded manner. Mice were housed under a 12:12 h light/dark cycle (lights off at 7:00 pm) with ad libitum access to food and water except during experimental sessions. All procedures were approved in advance by the Institutional Animal Care and Use Committee at the University of Florida and conducted in accordance with the 2011 NIH Guide for the Care and Use of Laboratory Animals.

### 4.4. Drug Preparation and Administration

Morphine and U50,488 (at final concentrations of 1.0 and 3.0 mg/mL) were dissolved in 0.9% sterile isotonic saline (saline) for testing. Gabapentin was dissolved in 5% dimethyl sulfoxide (DMSO)/95% saline at 5.0 mg/mL. **UVM147** (3.0 mg/mL), **RO-4-3** (3.0 mg/mL), **RO-5-3** (1.0, 3.0, 4.5 and 6.0 mg/mL), and **RO-7-3** (3.0, 4.5 and 6.0 mg/mL) were dissolved in 10% DMSO/10% Solutol/80% saline or 5% DMSO/95% saline. Morphine was administered intraperitoneally (i.p.) or subcutaneously (s.c.). Gabapentin and U50,488 were administered i.p., and all other drugs were administered s.c., in a volume of 250 µL per 25 g body weight.

### 4.5. Behavioral Assays

#### 4.5.1. Chronic Constriction Injury

CD-1 mice were used for the CCI assay. Mice were anesthetized with isoflurane as previously described [36,37,43,44,81]. A small skin incision was made along the exterior of the biceps femoris of the right hind paw. The muscle was separated using blunt forceps, and the right sciatic nerve was exposed. Two opposite-facing 0.1–10 µL pipette tips were placed under the sciatic nerve to facilitate passing two sutures 1 mm apart. The ligatures were loosely tied around the nerve and secured with two knots. The skin incision was closed using two 9 mm staples. After a 7-day recovery period, baseline von Frey testing confirmed the development of mechanical allodynia.

Von Frey testing was performed as described previously [36,38,39,83]. Filaments applying increasing force (0.4–6 g) were applied to the plantar surface of the hind paw, and withdrawal thresholds were measured. Thresholds reduced from baseline responses prior to surgery indicated neuropathic allodynia. Mice received vehicle (5% DMSO/95% saline, s.c.) or drugs, and withdrawal thresholds in both the contralateral and ipsilateral hind paws were recorded every 20 min thereafter up to 80 min post-injection. Each time point was measured in triplicate, with licking, shaking, or withdrawal considered a response. Gabapentin was administered 60 min prior to testing to avoid sedative confounds [41].

#### 4.5.2. Formalin Assay

Antinociception against an inflammatory stimulus was assessed using the formalin assay in C57BL/6J mice [37,40,84]. Drug or vehicle (5% DMSO/95% saline, s.c.) was administered 10 min prior to intraplantar injection of 5% formalin (2.5 µg in 15 µL) into the right hind paw. Time spent licking the injected paw was recorded in 5 min intervals for 60 min. The last 55 min of assessment was used to determine the inflammatory response stimulus. Data were analyzed as area under the curve (AUC), representing summed time mice spent licking the injected right hind paw.

#### 4.5.3. Measurement of Respiration and Locomotion

Respiration and spontaneous locomotion were measured using a computer-automated Comprehensive Lab Animal Monitoring System (CLAMS; Columbus Instruments, Columbus, OH, USA), as described previously [40,43,44,75,85]. Unrestrained C57BL/6J mice were habituated individually in sealed cages connected to the system for 60 min preceding testing for initial mouse readings. Mice were then administered s.c. drug or vehicle (5% DMSO/95% saline) and placed back into the sealed CLAMS testing units for 240 min. A built-in pressure transducer within the cages was used to measure respiration (breaths/min). Locomotion was measured via infrared photobeams located at the bottom of each cage. Ambulation was counted as the number of sequential photobeam breaks. All data are expressed as a percentage of vehicle response.

#### 4.5.4. Rotarod Assay

Motor coordination was assessed using the rotarod chambers (San Diego Instruments, San Diego, CA, USA) assay [43,44,76] with C57BL/6J mice. Seven training trials were performed where the last training trial was used as a baseline of performance. Vehicle (10% DMSO/10% Solutol/80% saline, s.c.), U50,488 (10 mg/kg, i.p.), **RO-5-3**, or **RO-7-3** (60 mg/kg, s.c.) were administered and then assessed in 10 min in accelerated speed trials (180 s max latency at 0–20 rpm) for 60 min. Latency to fall was measured in seconds. Data are reported as the average % difference from each mouse’s baseline latency reading. Reduced latencies to fall in the rotarod test suggest impaired motor coordination or sedation.

#### 4.5.5. Conditioned Place Preference

Automated, three-compartment place preference chambers (San Diego Instruments, San Diego, CA, USA) were used to evaluate conditioned place preference (CPP) in C57BL/6J mice. A two-day counterbalanced conditioning design [40,43,44,75,76,81,85] was employed, with initial place preference evaluation being tested 24 h prior to place conditioning. In initial preference testing, the mice were allowed free access to all compartments of the apparatus for 30 min, and total time spent in each compartment was recorded. For 2 days following initial preference evaluation, the mice were administered vehicle (5% DMSO/95% saline) and then confined to an outer compartment of the apparatus for 40 min. Four hours after saline administration, the mice were either administered morphine (10 mg/kg, i.p.), U50,488 (30 mg/kg, i.p.), **RO-5-3** (60 mg/kg, s.c.), or **RO-7-3** (45 mg/kg, s.c.) and then confined in the opposite outer compartment for 40 min. Conditioning restrictions were repeated precisely on day 2 of place conditioning. A total of 24 h after the second day of conditioning, final place preference was evaluated. Mice were allowed to roam freely between all chambers for 30 min. Data are expressed as the difference in time spent between the drug-paired and vehicle-paired compartments. Positive values represent conditioned preference, whereas negative values are considered conditioned aversion for the drug-paired side.

### 4.6. Statistical Analysis

Data are expressed as mean ± SEM. Analyses were performed using GraphPad Prism 10. Data were analyzed using either one-way or two-way ANOVA with the appropriate post hoc test (Dunnett’s, Sidak’s, or Tukey’s), where *p* < 0.05 was considered significant. Linear regression was used to determine ED_50_ values, and 95% confidence intervals of dose–response curves presented for the formalin assay. CPP data are represented as the difference between the time spent in the drug pair compartment and the vehicle paired compartment between pre- and post-conditioning. CLAMS data are reported as a % of matching vehicle responses. The rotarod data are expressed as the % change from baseline performance for each animal’s baseline response.

## 5. Conclusions

Three novel analogs **RO-4-3**, **RO-5-3**, and **RO-7-3** of the sigma-1-receptor selective ligand **UVM147** [31] showed high affinity and varying selectivity for S1R and S2R. Among the analogs, **RO-5-3** demonstrated both anti-allodynic and antinociceptive effects against inflammatory pain without locomotor effects, although it mildly depressed breathing and induced conditioned place aversion. Collectively, these data support the development of S1R ligands as therapeutics for the treatment of chronic pain.

## Figures and Tables

**Table 1:** Competition radioligand binding values (as *K_i_*, nM) of parent compound **UVM147** and its analogs **RO-4-3**, **RO-5-3**, and **RO-7-3** determined through the NIMH-PDSP screening program.

Receptors	RO-4-3		RO-5-3		RO-7-3		UVM147
	**p*K_i_* ± SEM**	***K_i_*** **(nM)**	**p*K_i_* ± SEM**	***K_i_*** **(nM)**	**p*K_i_* ± SEM**	***K_i_*** **(nM)**	***K_i_*** **(nM)**
**5-HT2BR**	5.58 ± 0.07	2633	-	-	-	-	231
**5-HT2CR**	5.63 ± 0.06	2364	-	-	-	-	-
**5-HT6R**	5.89 ± 0.05	1296	-	-	5.83 ± 0.09	1469	-
**5-HT7R**	5.39 ± 0.09	4116	-	-	6.33 ± 0.08	467	2765
**D1R**	6.15 ± 0.06	701	-	-	-	-	-
**D3R**	-	-	-	-	5.42 ± 0.06	3837	-
**D4R**	-	-	-	-	-	-	1283
**DAT**	-	-	-	-	-	-	2562
**SERT**	6.24 ± 0.05	572	-	-	5.91 ± 0.05	1235	-
**NET**	6.39 ± 0.08	407	-	-	-	-	-
**MOR**	5.96 ± 0.05	1084	5.52 ± 0.04	3048	5.83 ± 0.07	1490	-
**DOR**	-	-	-	-	5.6 ± 0.07	2489	-
**KOR**	5.96 ± 0.08	1107	5.84 ± 0.06	1457	6.2 ± 0.01	621	1707
**H1R**	6.1 ± 0.1	797	-	-	-	-	-
**H2R**	6.04 ± 0.05	915	6.16 ± 0.09	690	5.9 ± 0.06	1246	823
**α2A AR**	-	-	-	-	5.6 ± 0.1	2732	3364
**α2B AR**	-	-	5.99 ± 0.06	1010	-	-	-
**α2C AR**	-	-	6.35 ± 0.07	443	5.4 ± 0.1	3966	-
**S1R**	6.21 ± 0.7	620	7.57 ± 0.07	27	7.63 ± 0.08	24	8.6
**S2R**	6.73 ± 0.06	184	6.6 ± 0.08	250	6.5 ± 0.1	351	696

Dashes indicate *K_i_* value >10 μ M. Receptors listed are serotonin receptor subtypes: 5-HT2BR, 5-HT2CR, 5-HT6R, and 5-HT7R, with serotonin receptor subtypes 2B, 2C, 6, and 7, respectively. D1R, D3R, and D4R; dopamine receptor subtypes 1, 3, and 4, respectively. DAT, SERT, and NET are dopamine, serotonin, and norepinephrine transporter proteins, respectively. MOR, DOR, and KOR are mu, delta, and kappa opioid receptors, respectively. H1R and H2R are histamine H1 and H2 receptors. α2A AR, α2B AR, and α2C AR are alpha 2A, 2B, and 2C adrenergic receptors, respectively. S1R and S2R are sigma 1 and 2 receptors. The binding data for **UVM147** were collected from our published paper [31]. Detailed descriptions of radiolabeled and cold competitor ligands used and assay conditions can be found online at https://pdspdb.unc.edu/pdspweb/content/PDSP%20Protocols%20II%202013-03-28.pdf (accessed on 25 October 2025).

## Data Availability

The data presented in this study are available on request from the corresponding author due to privacy.

## Supplementary Materials

The following supporting information can be downloaded at: https://www.mdpi.com/article/10.3390/ph19010117/s1, ^1^H and ^13^C NMR spectra of UVM147, RO-4-3, RO-5-3, and RO-7-3; HRMS and LC data of RO-4-3, RO-5-3, and RO-7-3.

## Abbreviations

The following abbreviations are used in this manuscript:

ANOVA	Analysis of variance
CCI	Chronic Constriction Injury
CI	Confidence interval
CPP	Conditioned place preference
DMSO	Dimethyl sulfoxide
DOR	Delta opioid receptor
HATU	2-(1H-7-Azabenzotriazol-1-yl)-1,1,3,3-tetramethyluronium hexafluorophosphate
HOBt	Hydroxybenzotriazole
i.c.v.	Intracerebroventricular
i.p.	Intraperitoneal
ITR	Inverted terminal repeat
KOR	Kappa opioid receptor
MOR	Mu opioid receptor
ND	Not determined
NMDA	N-methyl-D-aspartate
RM	Repeated measures
SEM	Standard error of the mean
SR	Sigma receptor
S1R	Sigma-1 receptor
S2R	Sigma-2 receptor
SNC-80	(+)-4-[(αR)-α-((2S,5R)-4-Allyl-2,5-dimethyl-1-piperazinyl)-3-methoxybenzyl]-N,N-diethylbenzamide
TFA	Trifluoroacetic acid
U50,488	(±)-trans-3,4-Dichloro-N-methyl-N-[2-(1-pyrrolidinyl)cyclohexyl]benzeneacetamide
UHPLC	Ultrahigh performance liquid chromatography
WWTW	Warm-water tail withdrawal

## References

[1] Nicholson B., Verma S. (2004). Comorbidities in chronic neuropathic pain. Pain Med..

[2] Dosenovic S., Nikolic Z., Ivancev B., Jelicic Kadic A., Puljak L. (2019). Awareness and acceptability of Initiative on Methods, Measurement, and Pain Assessment in Clinical Trials core outcome set for chronic pain among surveyed neuropathic pain authors. J. Comp. Eff. Res..

[3] Colloca L., Ludman T., Bouhassira D., Baron R., Dickenson A.H., Yarnitsky D., Freeman R., Truini A., Attal N., Finnerup N.B. (2017). Neuropathic pain. Nat. Rev. Dis. Primers.

[4] van Hecke O., Austin S.K., Khan R.A., Smith B.H., Torrance N. (2014). Neuropathic pain in the general population: A systematic review of epidemiological studies. Pain.

[5] Kocot-Kępska M., Zajączkowska R., Mika J., Wordliczek J., Dobrogowski J., Przeklasa-Muszyńska A. (2021). Peripheral Mechanisms of Neuropathic Pain-the Role of Neuronal and Non-Neuronal Interactions and Their Implications for Topical Treatment of Neuropathic Pain. Pharmaceuticals.

[6] Felix R., Corzo-Lopez A., Sandoval A. (2025). Voltage-Gated Ion Channels in Neuropathic Pain Signaling. Life.

[7] Gao N., Li M., Wang W., Liu Z., Guo Y. (2024). The dual role of TRPV1 in peripheral neuropathic pain: Pain switches caused by its sensitization or desensitization. Front. Mol. Neurosci..

[8] Malcangio M. (2019). Role of the immune system in neuropathic pain. Scand. J. Pain.

[9] Cruccu G., Truini A. (2017). A review of neuropathic pain: From Guidelines to Clinical Practice. Pain Ther..

[10] Moisset X. (2024). Neuropathic pain: Evidence based recommendations. Presse Medicale.

[11] Finnerup N.B., Attal N., Haroutounian S., McNicol E., Baron R., Dworkin R.H., Gilron I., Haanpää M., Hansson P., Jensen T.S. (2015). Pharmacotherapy for neuropathic pain in adults: A systematic review and meta-analysis. Lancet Neurol..

[12] Volkow N., Benveniste H., McLellan A.T. (2018). Use and Misuse of Opioids in Chronic Pain. Ann. Rev. Med..

[13] Ray W.A., Chung C.P., Murray K.T., Hall K., Stein C.M. (2016). Prescription of long-acting opioids and mortality in patients with chronic noncancer pain. JAMA.

[14] Thouaye M., Yalcin I. (2023). Neuropathic pain: From actual pharmacological treatments to new therapeutic horizons. Pharmacol. Ther..

[15] Bouhassira D., Attal N. (2018). Emerging therapies for neuropathic pain: New molecules or new indications for old treatments?. Pain.

[16] Linciano P., Rossino G., Listro R., Rossi D., Collina S. (2020). Sigma-1 receptor antagonists: Promising players in fighting neuropathic pain. Pharm. Pat. Anal..

[17] Sánchez-Fernández C., Entrena J.M., Baeyens J.M., Cobos E.J. (2017). Sigma-1 receptor antagonists: A new class of neuromodulatory analgesics. Sigma Receptors: Their Role in Disease and as Therapeutic Targets; Advances in Experimental Medicine and Biology.

[18] Martin W.R., Eades C.G., Thompson J.A., Huppler R.E., Gilbert P.E. (1976). The effects of morphine- and nalorphine- like drugs in the nondependent and morphine-dependent chronic spinal dog. J. Pharmacol. Exp. Ther..

[19] Hanner M., Moebius F.F., Flandorfer A., Knaus H.G., Striessnig J., Kempner E., Glossmann H. (1996). Purification, molecular cloning, and expression of the mammalian sigma1-binding site. Proc. Natl. Acad. Sci. USA.

[20] Alon A., Schmidt H.R., Wood M.D., Sahn J.J., Martin S.F., Kruse A.C. (2017). Identification of the gene that codes for the σ2 receptor. Proc. Natl. Acad. Sci. USA.

[21] Kim H.W., Roh D.H., Yoon S.Y., Seo H.S., Kwon Y.B., Han H.J., Kim K.W., Beitz A.J., Lee J.H. (2008). Activation of the spinal sigma-1 receptor enhances NMDA-induced pain via PKC- and PKA-dependent phosphorylation of the NR1 subunit in mice. Br. J. Pharmacol..

[22] Roh D.H., Choi S.R., Yoon S.Y., Kang S.Y., Moon J.Y., Kwon S.G., Han H.J., Beitz A.J., Lee J.H. (2011). Spinal neuronal NOS activation mediates sigma-1 receptor-induced mechanical and thermal hypersensitivity in mice: Involvement of PKC-dependent GluN1 phosphorylation. Br. J. Pharmacol..

[23] Shin S.M., Wang F., Qiu C., Itson-Zoske B., Hogan Q.H., Yu H. (2022). Sigma-1 receptor activity in primary sensory neurons is a critical driver of neuropathic pain. Gene Ther..

[24] Romero L., Zamanillo D., Nadal X., Sánchez-Arroyos R., Rivera-Arconada I., Dordal A., Montero A., Muro A., Bura A., Segalés C. (2012). Pharmacological properties of S1RA, a new sigma-1 receptor antagonist that inhibits neuropathic pain and activity-induced spinal sensitization. Br. J. Pharmacol..

[25] Bravo-Caparrós I., Perazzoli G., Yeste S., Cikes D., Baeyens J.M., Cobos E.J., Nieto F.R. (2019). Sigma-1 receptor inhibition reduces neuropathic pain induced by partial sciatic nerve transection in mice by opioid-dependent and -independent mechanisms. Front. Pharmacol..

[26] Ruiz-Cantero M.C., González-Cano R., Tejada M.Á., Santos-Caballero M., Perazzoli G., Nieto F.R., Cobos E.J. (2021). Sigma-1 receptor: A drug target for the modulation of neuroimmune and neuroglial interactions during chronic pain. Pharmacol. Res..

[27] Merlos M., Romero L., Zamanillo D., Plata-Salamán C., Vela J.M. (2017). Sigma-1 receptor and pain. Sigma Proteins: Evolution of the Concept of Sigma Receptors; Handbook of Experimental Pharmacology.

[28] Bruna J., Videla S., Argyriou A.A., Velasco R., Villoria J., Santos C., Nadal C., Cavaletti G., Alberti P., Briani C. (2018). Efficacy of a novel sigma-1 receptor antagonist for oxaliplatin-induced neuropathy: A randomized, double-blind, placebo-controlled phase IIa clinical trial. Neurotherapeutics.

[29] Bruna J., Velasco R. (2018). Sigma-1 receptor: A new player in neuroprotection against chemotherapy-induced peripheral neuropathy. Neural Regen. Res..

[30] Entrena J.M., Cobos E.J., Nieto F.R., Cendán C.M., Gris G., Del Pozo E., Zamanillo D., Baeyens J.M. (2009). Sigma-1 receptors are essential for capsaicin-induced mechanical hypersensitivity: Studies with selective sigma-1 ligands and sigma-1 knockout mice. Pain.

[31] Uprety R., Váradi A., Allaoa A., Redel-Traub G.N., Palmer T.C., Feinberg E.N., Ferris A.C., Pande V.S., Pasternak G.W., Majumdar S. (2019). Synthesis of spiro-2,6-dioxopiperazine and spiro-2,6-dioxopyrazine scaffolds using amino acids in a three-component reaction to generate potential Sigma-1 (σ1) receptor selective ligands. Eur. J. Med. Chem..

[32] Besnard J., Ruda G.F., Setola V., Abecassis K., Rodriguiz R.M., Huang X.P., Norval S., Sassano M.F., Shin A.I., Webster L.A. (2012). Automated design of ligands to polypharmacological profiles. Nature.

[33] Váradi A., Palmer T.C., Notis P.R., Redel-Traub G.N., Afonin D., Subrath J.J., Pasternak G.W., Hu C., Sharma I., Majumdar S. (2014). Three-component coupling approach for the synthesis of diverse heterocycles utilizing reactive nitrilium trapping. Org. Lett..

[34] Váradi A., Palmer T.C., Notis Dardashti R., Majumdar S. (2015). Isocyanide-based multicomponent reactions for the synthesis of heterocycles. Molecules.

[35] John C.S., Vilner B.J., Bowen W.D. (1994). Synthesis and characterization of [^125^I]-N-(N-benzylpiperidin-4-yl)-4- iodobenzamide, a new sigma receptor radiopharmaceutical: High-affinity binding to MCF-7 breast tumor cells. J. Med. Chem..

[36] Kovács F., Huliák I., Árva H., Kocsis M., Kiricsi M., Frank É. (2025). Aromatic scaffold-integrated hybrids of estradiol and benzoxazol-2-ones: Synthesis and in vitro anticancer activity of N-substituted regioisomeric pairs. RSC Adv..

[37] Hoot M.R., Sim-Selley L.J., Poklis J.L., Abdullah R.A., Scoggins K.L., Selley D.E., Dewey W.L. (2010). Chronic constriction injury reduces cannabinoid receptor 1 activity in the rostral anterior cingulate cortex of mice. Brain Res..

[38] Bennett G.J., Xie Y.K. (1988). A peripheral mononeuropathy in rat that produces disorders of pain sensation like those seen in man. Pain.

[39] Xu M., Petraschka M., McLaughlin J.P., Westenbroek R.E., Caron M.G., Lefkowitz R.J., Czyzyk T.A., Pintar J.E., Terman G.W., Chavkin C. (2004). Neuropathic pain activates the endogenous kappa opioid system in mouse spinal cord and induces opioid receptor tolerance. J. Neurosci..

[40] Cirino T.J., Eans S.O., Medina J.M., Wilson L.L., Mottinelli M., Intagliata S., McCurdy C.R., McLaughlin J.P. (2019). Characterization of sigma 1 receptor antagonist CM-304 and its analog, AZ-66: Novel therapeutics against allodynia and induced pain. Front. Pharmacol..

[41] Gilron I. (2007). Gabapentin and pregabalin for chronic neuropathic and early postsurgical pain: Current evidence and future directions. Curr. Opin. Anaesthesiol..

[42] De Vry J., Kuhl E., Franken-Kunkel P., Eckel G. (2004). Pharmacological characterization of the chronic constriction injury model of neuropathic pain. Eur. J. Pharmacol..

[43] Wilson L.L., Alleyne A.R., Eans S.O., Cirino T.J., Stacy H.M., Mottinelli M., Intagliata S., McCurdy C.R., McLaughlin J.P. (2022). Characterization of CM-398, a novel, selective sigma-2 receptor ligand, as a potential therapeutic for neuropathic pain. Molecules.

[44] Wilson L.L., Eans S.O., Ramadan-Siraj I., Modica M.N., Romeo G., Intagliata S., McLaughlin J.P. (2022). Examination of the novel sigma-1 receptor antagonist, SI 1/28, for antinociceptive and anti-allodynic efficacy against multiple types of nociception with fewer liabilities of use. Int. J. Mol. Sci..

[45] Roh D.H., Kim H.W., Yoon S.Y., Seo H.S., Kwon Y.B., Kim K.W., Han H.J., Beitz A.J., Na H.S., Lee J.H. (2008). Intrathecal injection of the sigma(1) receptor antagonist BD1047 blocks both mechanical allodynia and increases in spinal NR1 expression during the induction phase of rodent neuropathic pain. Anesthesiology.

[46] Barcelon E.E., Cho W.H., Jun S.B., Lee S.J. (2019). Brain Microglial Activation in Chronic Pain-Associated Affective Disorder. Front. Neurosci..

[47] Choi J.G., Choi S.R., Kang D.W., Kim J., Park J.B., Lee J.H., Kim H.W. (2022). Sigma-1 receptor increases intracellular calcium in cultured astrocytes and contributes to mechanical allodynia in a model of neuropathic pain. Brain Res. Bull..

[48] Vidal-Torres A., Fernández-Pastor B., Carceller A., Vela J.M., Merlos M., Zamanillo D. (2014). Effects of the selective sigma-1 receptor antagonist S1RA on formalin-induced pain behavior and neurotransmitter release in the spinal cord in rats. J. Neurochem..

[49] McCall W.D., Tanner K.D., Levine J.D. (1996). Formalin induces biphasic activity in C-fibers in the rat. Neurosci. Lett..

[50] Wheeler-Aceto H., Cowan A. (1991). Standardization of the rat paw formalin test for the evaluation of analgesics. Psychopharmacology.

[51] Abbott F.V., Franklin K.B.J., Westbrook F.R. (1995). The formalin test: Scoring properties of the first and second phases of the pain response in rats. Pain.

[52] Baba H., Doubell T.P., Woolf C.J. (1999). Peripheral inflammation facilitates Abeta fiber-mediated synaptic input to the substantia gelatinosa of the adult rat spinal cord. J. Neurosci..

[53] Zamanillo D., Romero L., Merlos M., Vela J.M. (2013). Sigma 1 receptor: A new therapeutic target for pain. Eur. J. Pharmacol..

[54] Drews E., Zimmer A. (2009). Central sensitization needs sigma receptors. Pain.

[55] Puente B., Nadal X., Portillo-Salido E., Sánchez-Arroyos R., Ovalle S., Palacios G., Muro A., Romero L., Entrena J.M., Baeyens J.M. (2009). Sigma-1 receptors regulate activity-induced spinal sensitization and neuropathic pain after peripheral nerve injury. Pain.

[56] Boom M., Niesters M., Sarton E., Aarts L., Smith T.W., Dahan A. (2012). Non-analgesic effects of opioids: Opioid-induced respiratory depression. Curr. Pharm. Des..

[57] Evoy K.E., Morrison M.D., Saklad S.R. (2017). Abuse and misuse of pregabalin and gabapentin. Drugs.

[58] Flórez J., Delgado G., Armijo J.A. (1972). Adrenergic and serotonergic mechanisms in morphine-induced respiratory depression. Psychopharmacologia.

[59] Manzanedo C., Aguilar M.A., Miñarro J. (1999). The effects of dopamine D2 and D3 antagonists on spontaneous motor activity and morphine-induced hyperactivity in male mice. Psychopharmacology.

[60] Imam M.Z., Kuo A., Smith M.T. (2020). Countering opioid-induced respiratory depression by non-opioids that are respiratory stimulants. F1000Research.

[61] Mei J., Pasternak G.W. (2007). Modulation of brainstem opiate analgesia in the rat by sigma 1 receptors: A microinjection study. J. Pharmacol. Exp. Ther..

[62] Zhang L.S., Wang J., Chen J.C., Tao Y.M., Wang Y.H., Xu X.J., Chen J., Xu Y.G., Xi T., Hu X.W. (2015). Novel κ-opioid receptor agonist MB-1C-OH produces potent analgesia with less depression and sedation. Acta Pharmacol. Sin..

[63] Dunn A.D., Reed B., Guariglia C., Dunn A.M., Hillman J.M., Kreek M.J. (2018). Structurally related kappa opioid receptor agonists with substantial differential signaling bias: Neuroendocrine and behavioral effects in C57BL6 mice. Int. J. Neuropsychopharmacol..

[64] Seth P., Scholl L., Rudd R.A., Bacon S. (2018). Overdose deaths involving opioids, cocaine, and psychostimulants—United States, 2015-2016. MMWR Morb. Mortal. Wkly. Rep..

[65] Seth P., Rudd R.A., Noonan R.K., Haegerich T.M. (2018). Quantifying the epidemic of prescription opioid overdose deaths. Am. J. Public Health.

[66] McKendrick G., Graziane N.M. (2020). Drug-induced conditioned place preference and its practical use in substance use disorder research. Front. Behav. Neurosci..

[67] Vezina P., Stewart J. (1987). Morphine conditioned place preference and locomo-tion: The effect of confinement during training. Psychopharmacology.

[68] Shippenberg T.S., Herz A. (1987). Place preference conditioning reveals the involvement of D1-dopamine receptors in the motivational properties of mu- and kappa-opioid agonists. Brain Res..

[69] Abadias M., Escriche M., Vaqué A., Sust M., Encina G. (2013). Safety, tolerability and pharmacokinetics of single and multiple doses of a novel sigma-1 receptor antagonist in three randomized phase I studies. Br. J. Clin. Pharmacol..

[70] Fraser M.R., Levine M. (2019). A Public Health Guide to Ending the Opioid Epidemic. A Comprehensive Approach to Addressing the Opioid Crisis.

[71] Gálvez R., Mayoral V., Cebrecos J., Medel F.J., Morte A., Sust M., Vaqué A., Montes-Pérez A., Neira-Reina F., Cánovas L. (2025). E-52862-A selective sigma-1 receptor antagonist, in peripheral neuropathic pain: Two randomized, double-blind, phase 2 studies in patients with chronic postsurgical pain and painful diabetic neuropathy. Eur. J. Pain.

[72] Wong E.H., Knight A.R., Woodruff G.N. (1988). [^3^H]MK-801 labels a site on the N-methyl-D-aspartate receptor channel complex in rat brain membranes. J. Neurochem..

[73] Palmer C.P., Aydar E., Jackson M.B., Su T.-P., Matsumoto R.R., Bowen W.D. (2007). σ Receptor modulation of ion channels. Sigma Receptors: Chemistry, Cell Biology and Clinical Implications.

[74] Merlos M., Burgueño J., Portillo-Salido E., Plata-Salamán C.R., Vela J.M. (2017). Pharmacological modulation of the sigma 1 receptor and the treatment of pain. Sigma Receptors: Their Role in Disease and as Therapeutic Targets; Advances in Experimental Medicine and Biology.

[75] Reilley K.J., Giulianotti M., Dooley C.T., Nefzi A., McLaughlin J.P., Houghten R.A. (2010). Identification of two novel, potent, low-liability antinociceptive compounds from the direct in vivo screening of a large mixture-based combinatorial library. AAPS J..

[76] Eans S.O., Ganno M.L., Mizrachi E., Houghten R.A., Dooley C.T., McLaughlin J.P., Nefzi A. (2015). Parallel synthesis of hexahydrodiimidazodiazepines heterocyclic peptidomimetics and their in vitro and in vivo activities at μ (MOR), δ (DOR), and κ (KOR) opioid receptors. J. Med. Chem..

[77] Brabant C., Quertemont E., Tirelli E. (2005). Influence of the dose and the num-ber of drug-context pairings on the magnitude and the long-lasting retention of cocaine-induced conditioned place preference in C57BL/6J mice. Psychopharmacology.

[78] Orsini C., Bonito-Oliva A., Conversi D., Cabib S. (2005). Susceptibility to con-ditioned place preference induced by addictive drugs in mice of the C57BL/6 and DBA/2 inbred strains. Psychopharmacology.

[79] Feehan A.K., Morgenweck J., Zhang X., Amgott-Kwan A.T., Zadina J.E. (2017). Novel Endomorphin Analogs Are More Potent and Longer-Lasting Analgesics in Neuropathic, Inflammatory, Postoperative, and Visceral Pain Relative to Morphine. J. Pain.

[80] Intagliata S., Sharma A., King T.I., Mesangeau C., Seminerio M., Chin F.T., Wilson L.L., Matsumoto R.R., McLaughlin J.P., Avery B.A. (2020). Discovery of a highly selective sigma-2 receptor ligand, 1-(4-(6,7-Dimethoxy-3,4-dihydroisoquinolin-2(1H)-yl)butyl)-3-methyl-1H-benzo[d]imidazol-2(3H)-one (CM398), with drug-like properties and atinociceptive effects In Vivo. AAPS J..

[81] Varga B.R., Bernhard S.M., El Daibani A., Zaidi S.A., Lam J.H., Aguilar J., Appourchaux K., Nazarova A.L., Kouvelis A., Shinouchi R. (2025). Structure-guided design of partial agonists at an opioid receptor. Nat. Comm..

[82] Kilkenny C., Browne W.J., Cuthi I., Emerson M., Altman D.G. (2012). Improving bioscience research reporting: The ARRIVE guidelines for reporting animal research. Vet. Clin. Pathol..

[83] Cheng H.Y., Pitcher G.M., Laviolette S.R., Whishaw I.Q., Tong K.I., Kockeritz L.K., Wada T., Joza N.A., Crackower M., Goncalves J. (2002). DREAM is a critical transcriptional repressor for pain modulation. Cell.

[84] Faouzi A., Wang H., Zaidi S.A., DiBerto J.F., Che T., Qu Q., Robertson M.J., Madasu M., El-Daibani A.E., Varga B.R. (2023). Structure-based design of bitopic ligands for the μ-opioid receptor. Nature.

[85] Varga B.R., Uprety R., Paul B., Knoll A., Ramos-Gonzalez N., Appourchaux K., Taylor T., Uzrek B., Shinouchi R., Eans S.O. (2025). Synthesis and pharmacology of a morphinan derived dual mu-kappa opioid receptor agonist analgesic. ACS Chem. Neurosci..

