# Supplementary material for: Characterization of Novel Sigma Receptor Ligands Derived from Multicomponent Reactions as Efficacious Treatments for Neuropathic Pain
Source: Pharmaceuticals (Basel). 2026 Jan 8;19(1):117. doi: 10.3390/ph19010117 (PMC12844632; doi:10.3390/ph19010117)
Supplement: Supplementary file 1 [file pharmaceuticals-19-00117-s001.zip › pharmaceuticals-4013243-supplementary.pdf]

Supplementary Information

Characterization of Novel Sigma Receptor Ligands Derived from Multicomponent Reactions as Efficacious Treatments for Neuropathic Pain

Ryosuke Shinouchi [1], Bengisu Turgutalp [2], Rohini S. Ople [2], Shainnel O. Eans [1], Ashai K. Williams [1], Haylee R. Hammond [1], Andras Varadi [3], Rebecca Notis Dardashti [3], Susruta Majumdar [2] and Jay P. McLaughlin [1,*]

1 Department of Cellular and Systems Pharmacology, College of Pharmacy, University of Florida, Gainesville, FL 32610, USA; r.shinouchi@ufl.edu (R.S.); shaieans@cop.ufl.edu (S.O.E.); williamsashai@ufl.edu (A.K.W.); hhammond@ufl.edu (H.R.H.)

2 Center for Clinical Pharmacology and Department of Anesthesiology, Washington University Pain Center and Washington University School of Medicine, St. Louis, MO 63131, USA; bengisut@wustl.edu (B.T.); rohiniopale@gmail.com (R.S.O.); susrutam@email.wustl.edu (S.M.)

3 Department of Neurology, Molecular Pharmacology and Chemistry, Memorial Sloan Kettering Cancer Center, New York, NY 10065, USA; uvamortifera@gmail.com (A.V.); ricki-notis@gmail.com (R.N.D.)

* Correspondence: jmclaughlin@cop.ufl.edu; Tel.: +1-352-273-7207

Table of Contents:

**^{1}H and ^{13}C NMR spectra of UVM147**	**S1**
**^{1}H and ^{13}C NMR spectra of RO-4-3**	**S2**
**^{1}H and ^{13}C NMR spectra of RO-5-3**	**S3**
**^{1}H and ^{13}C NMR spectra of RO-7-3**	**S4**
**HPLC data of RO-4-3, RO-5-3, and RO-7-3**	**S5-S7**
**HRMS data of RO-4-3, RO-5-3, and RO-7-3**	**S8-S13**

^{1}H-NMR (400 MHz; $CDCl_3$) and ^{13}C-NMR (100 MHz; $CDCl_3$) spectra of **UVM147** (CAS No: 2262446-43-3)

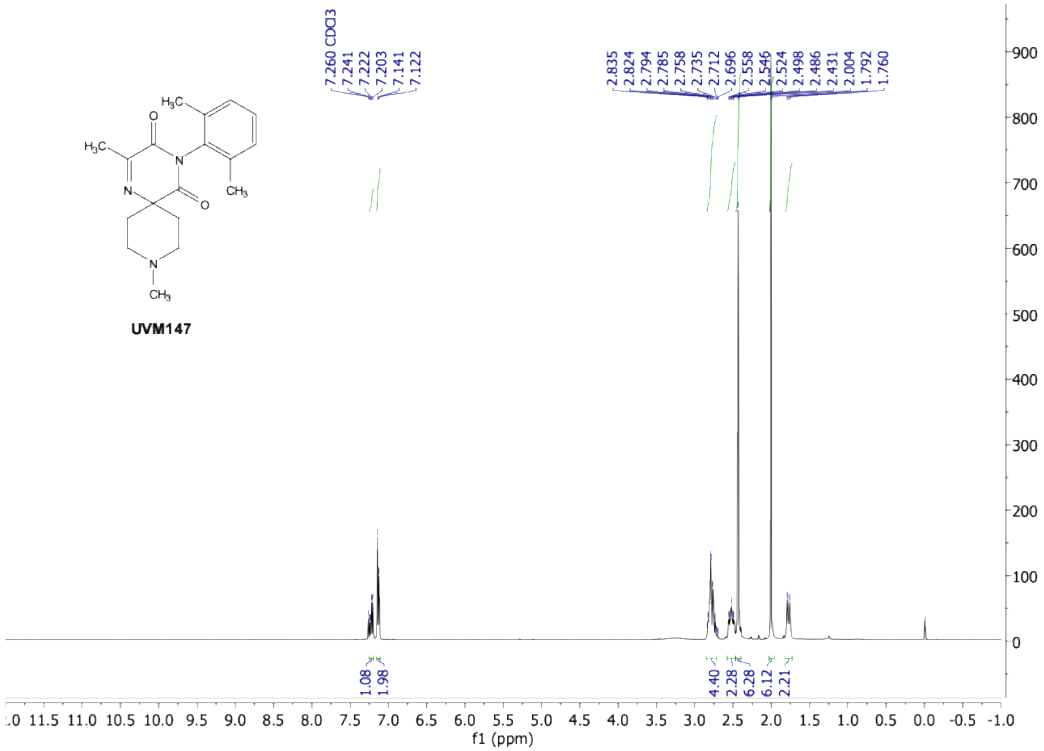

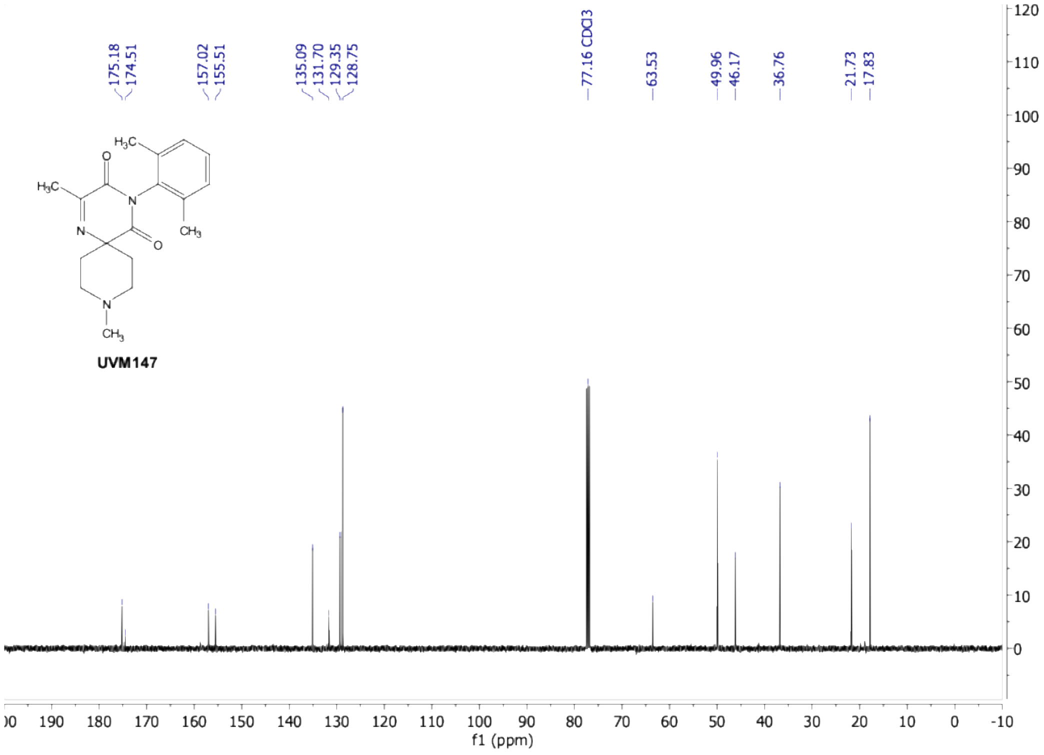

^{1}H-NMR (400 MHz; $CDCl_3$) and ^{13}C-NMR (100 MHz; $CDCl_3$) spectra of **RO-4-3**

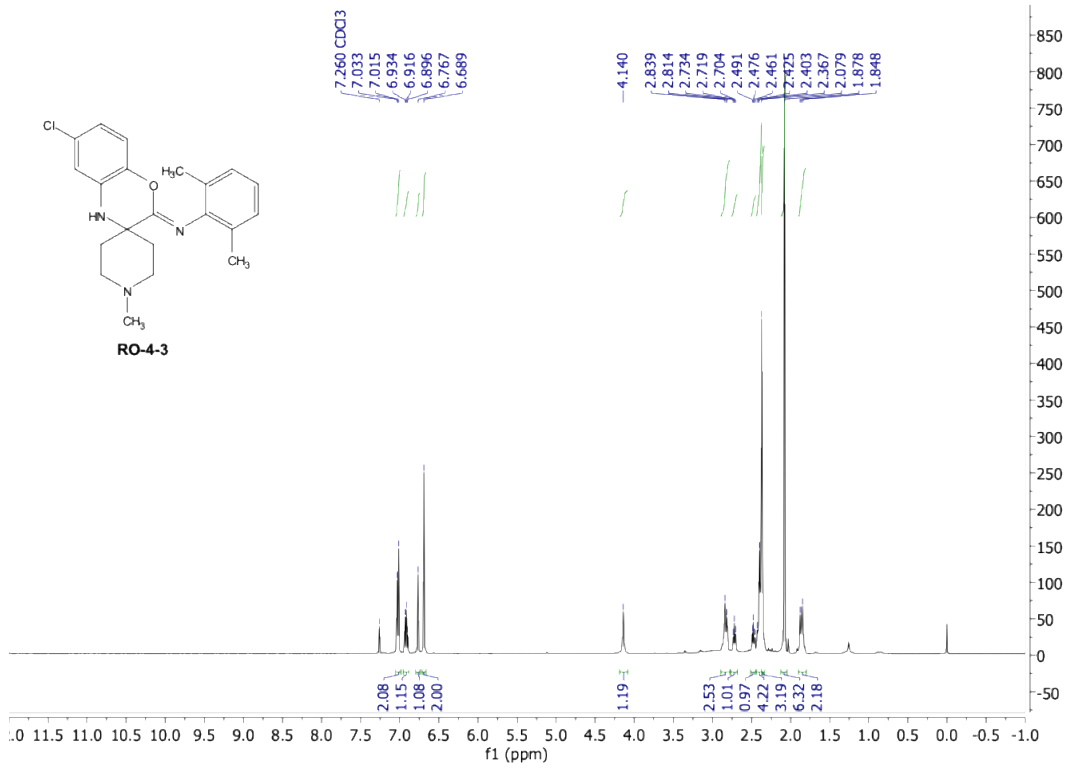

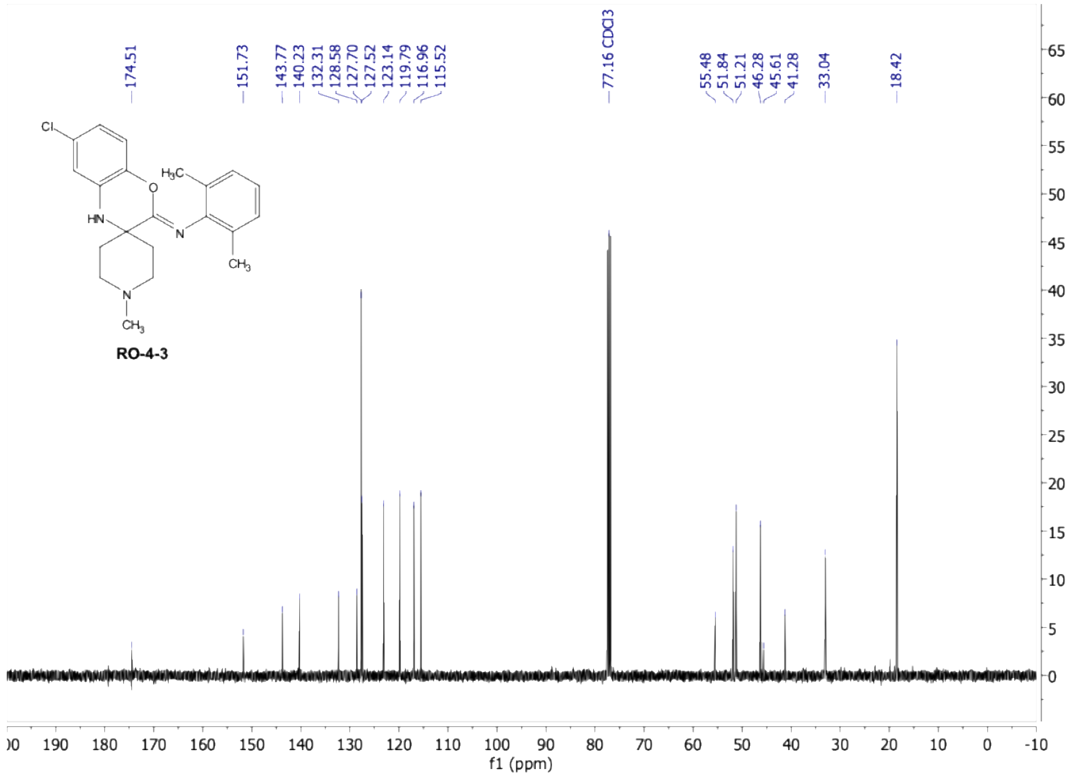

^{1}H-NMR (400 MHz; $CDCl_3$) and ^{13}C-NMR (100 MHz; $CDCl_3$) spectra of **RO-5-3**

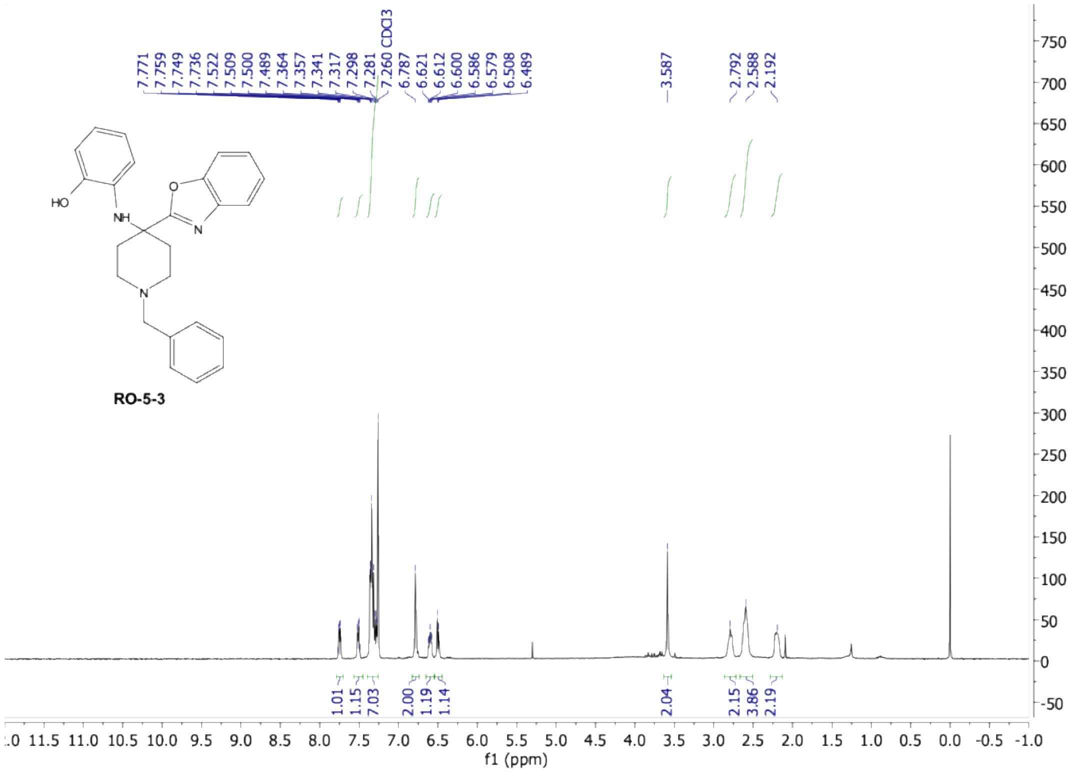

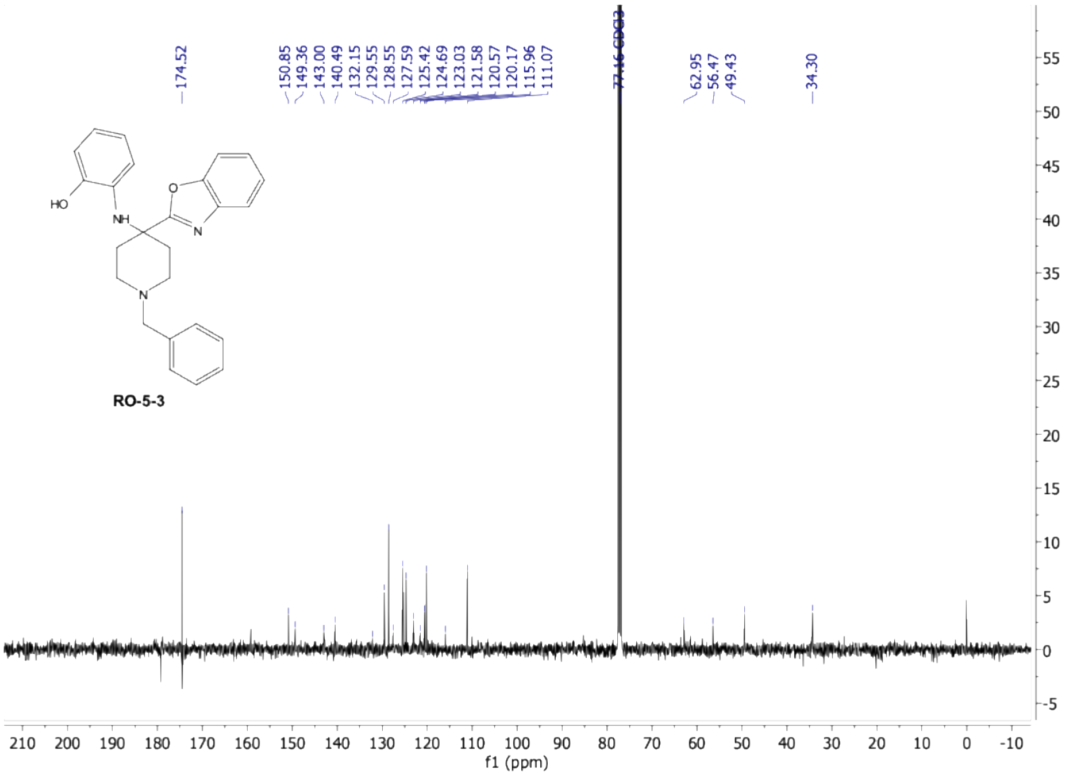

^{1}H-NMR (400 MHz; $CDCl_3$) and ^{13}C-NMR (100 MHz; $CDCl_3$) spectra of **RO-7-3**

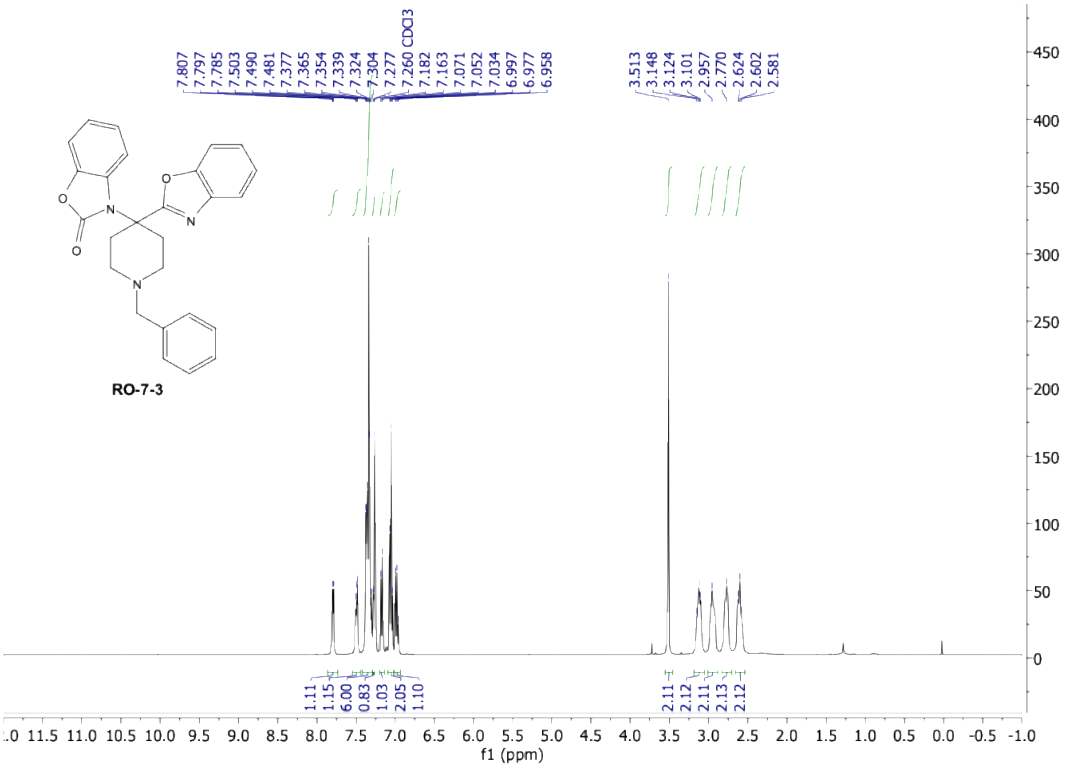

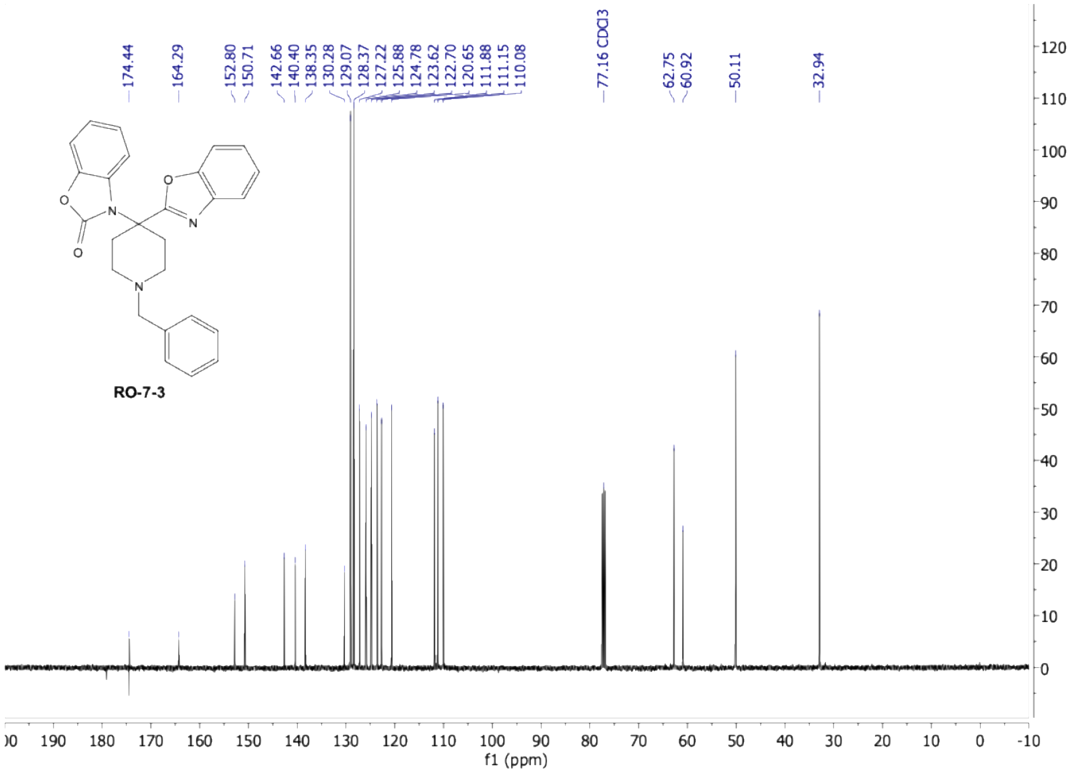

HPLC traces of RO 4-3

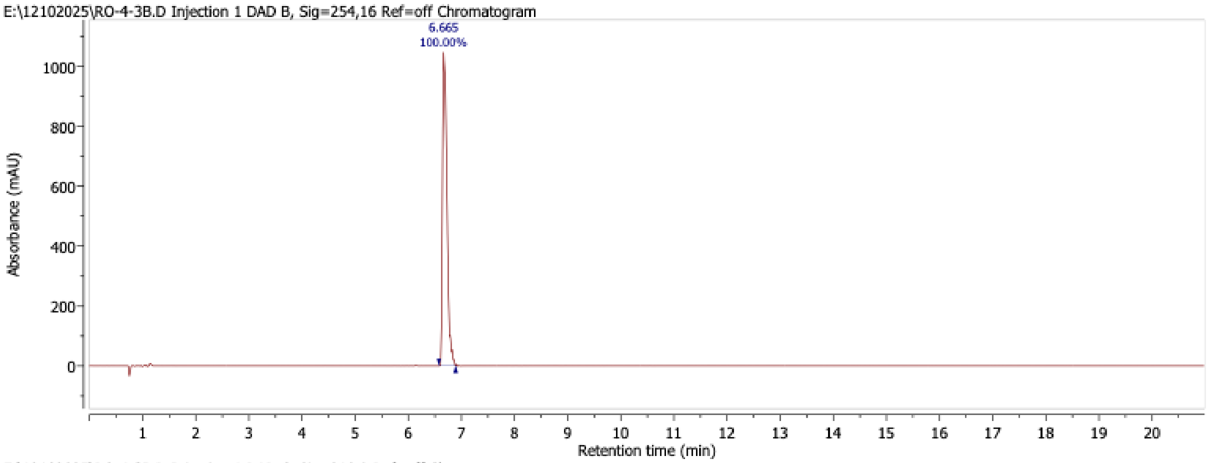

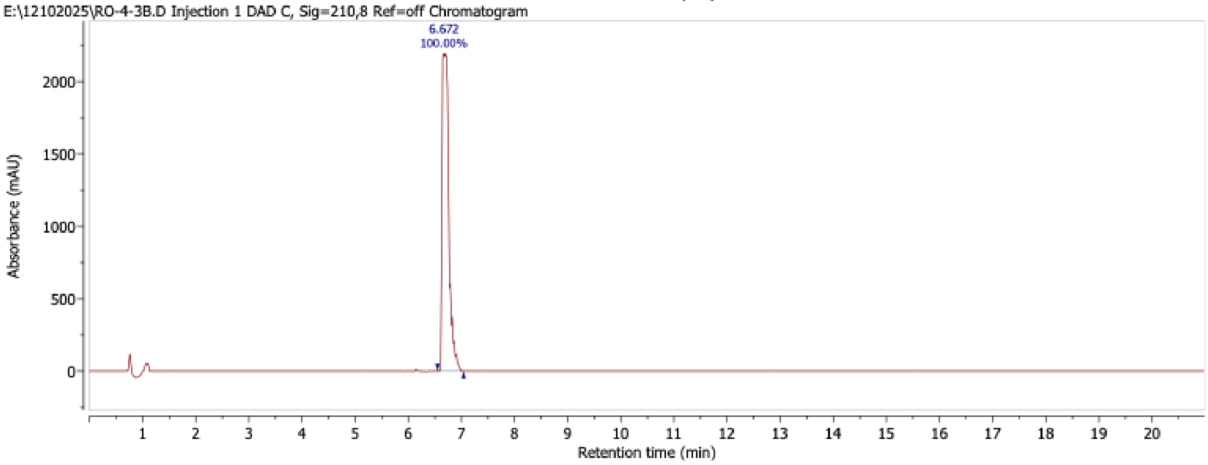

HPLC trace of RO5-3

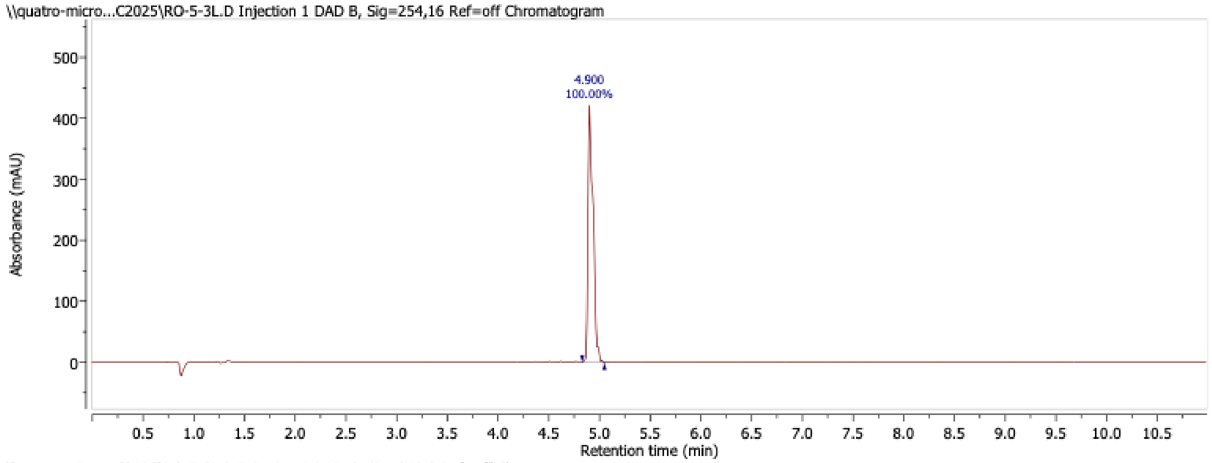

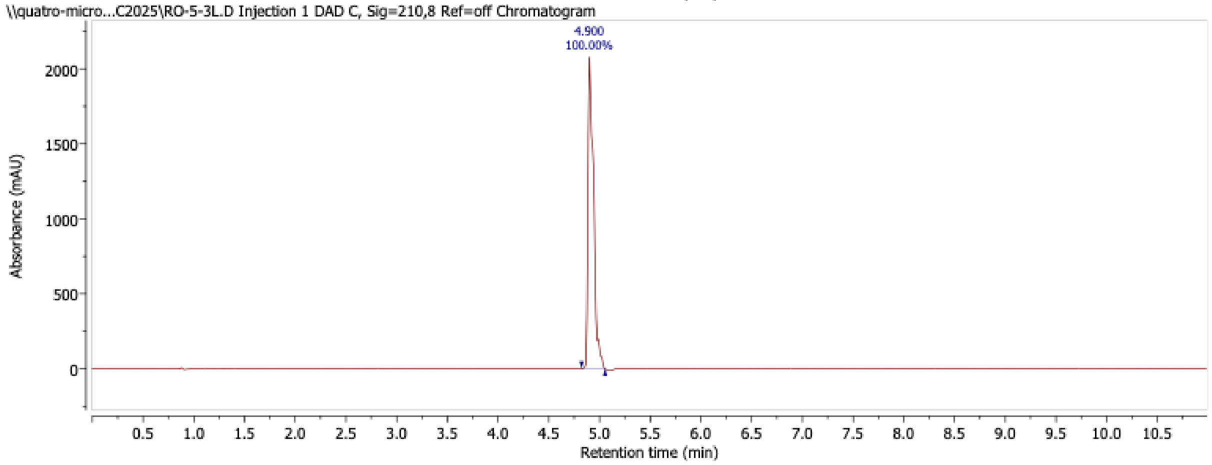

HPLC trace of RO7-3

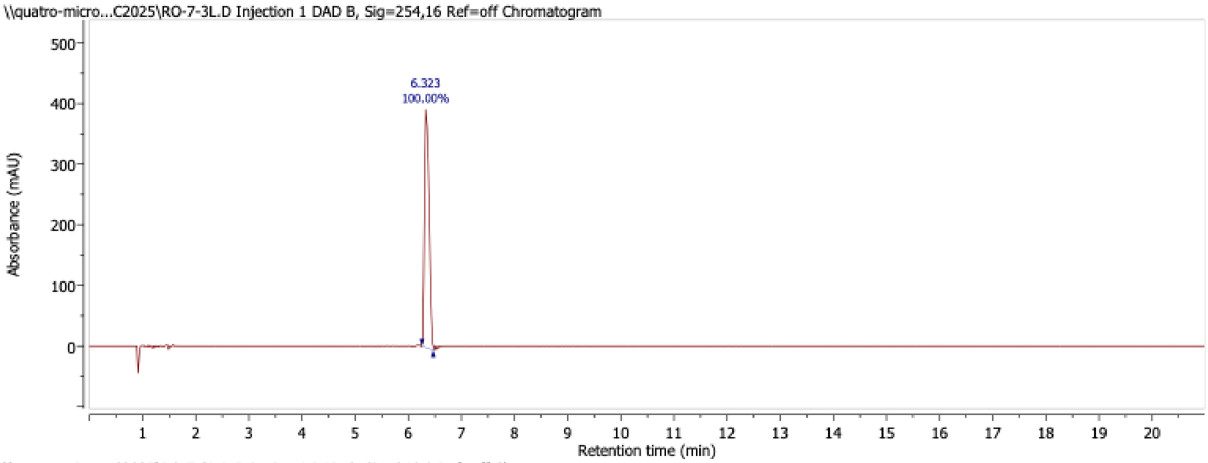

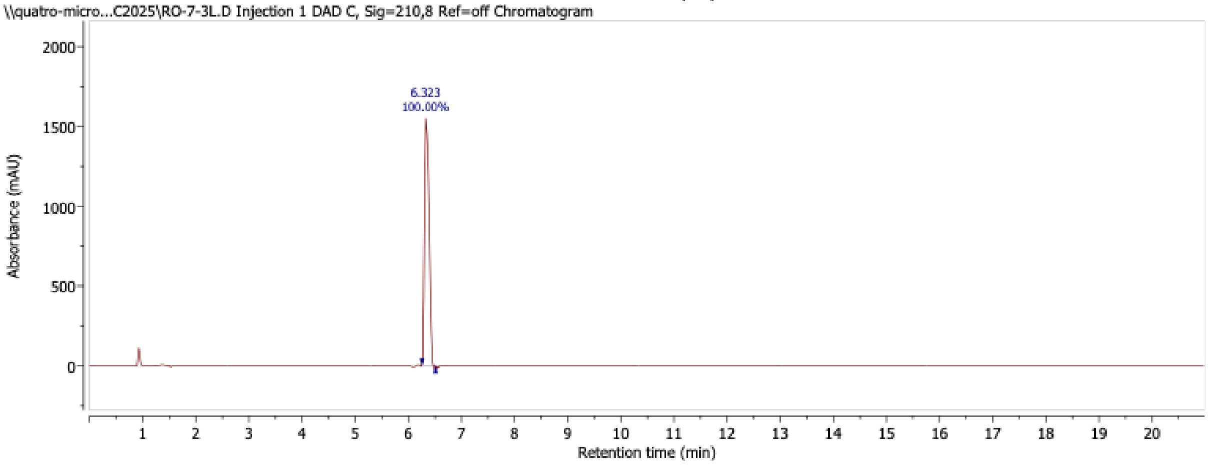

Mass Spectrum List Report

**Analysis Info**		Acquisition Date	10/21/2025 5:47:02 PM
Analysis Name	E:\MS1 COSMIC\2025\Data_20251021\RO-4-3_pos_000001.d		
Method		Operator	Admin
Sample Name	RO-4-3	Instrument	solariX XR
Comment	RO-4-3 in MeOH C21H24ClN3O H+		

Sample Name RO-4-3 in MeOH

Exact Mass of C21H24ClN3O H+ = 370.168067 m/z

Mass Observed = 370.167943 m/z

Difference < 1.0 ppm

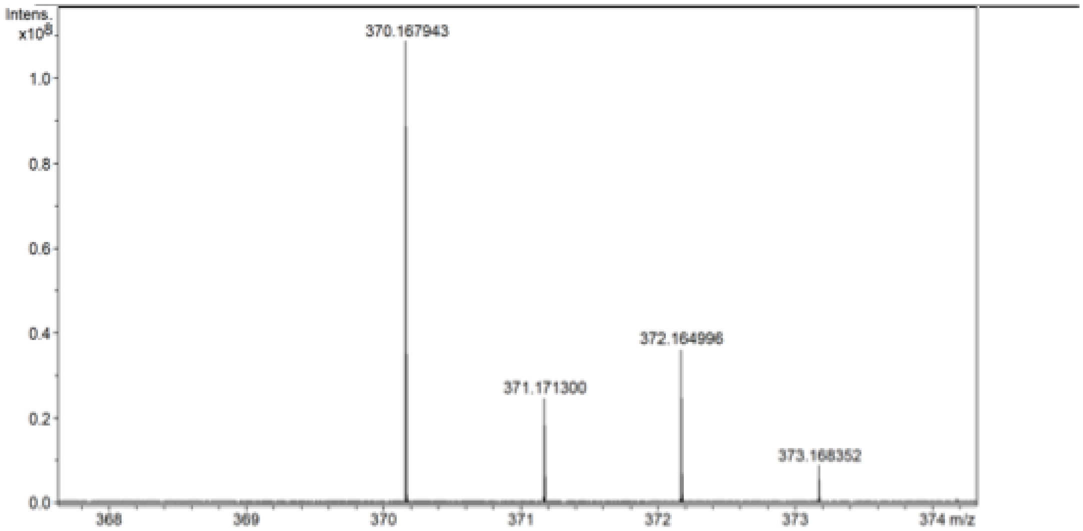

#	m/z	I
1	187.737989	528907
2	187.738229	641334
3	197.618461	515365
4	197.618757	1065628
5	197.618955	574867
6	197.619241	736143
7	208.597731	1004353
8	208.597993	526665
9	209.893887	505628
10	213.142219	454942
11	217.891788	457050
12	218.221077	692465
13	220.868037	1544703
14	220.868409	964959
15	232.416667	461318
16	234.671884	1223707
17	250.316759	802681
18	268.195978	841391
19	268.196747	774863
20	280.569173	1483603

Mass Spectrum List Report

#	m/z	I
21	288.826375	1833971
22	288.826942	1039411
23	312.895070	1156061
24	341.338914	616824
25	341.339582	1173048
26	345.178324	5701550
27	346.181653	1024847
28	370.166544	2059138
29	370.166925	3327874
30	370.167294	8010627
31	370.167943	108850048
32	370.168601	8971138
33	370.169322	2501507
34	370.169708	1553282
35	371.164988	1248940
36	371.171300	24910764
37	371.174277	578156
38	372.164330	2995157
39	372.164996	36535252
40	372.165655	3058645
41	372.174664	2821589
42	373.168352	8820733
43	374.171704	1028262
44	375.472533	1342426
45	381.253489	1006265
46	386.162842	770670
47	391.229760	767649
48	392.149893	6877889
49	393.153259	1717219
50	394.146954	2669317
51	395.150306	619559
52	400.201827	2255310
53	401.205142	614510
54	413.211697	735720
55	417.190876	1339874
56	417.191719	1337698
57	469.338755	1589679
58	469.340614	684079
59	536.385844	1723256
60	618.765139	539388
61	625.780609	1366235
62	654.649950	756279
63	750.933752	880598
64	761.310512	706011
65	762.313994	810764
66	763.307986	665403
67	938.658395	945924
68	950.943941	618852

Mass Spectrum List Report

Analysis Info		Acquisition Date	10/21/2025 5:38:41 PM
Analysis Name	E:\MS1 COSMIC\2025\Data_20251021\RO-5-3_pos_000001.d		
Method		Operator	Admin
Sample Name	RO-5-3	Instrument	solariX XR
Comment	RO-5-3 in MeOH C25H25N3O2 H+		

Sample Name RO-5-3 in MeOH

Exact Mass of C25H25N3O2 H+ = 400.201954 m/z

Mass Observed = 400.201827 m/z

Difference < 1.0 ppm

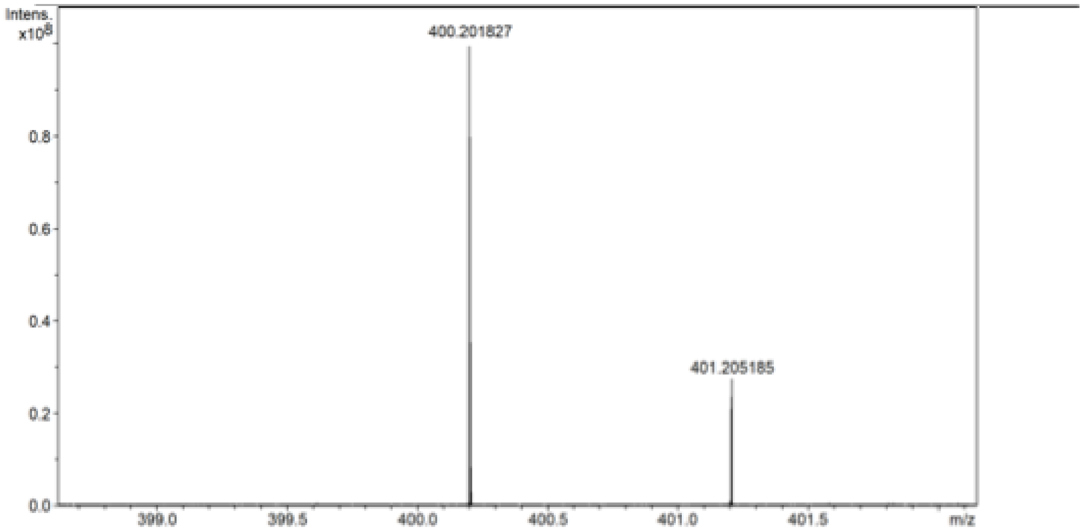

#	m/z	I
1	187.728883	744557
2	197.609152	1498684
3	197.609369	1042369
4	199.918629	478713
5	208.587038	700919
6	208.587650	716955
7	218.221095	808521
8	220.857312	1396306
9	234.660099	584754
10	234.660530	1637698
11	245.297083	495366
12	250.304257	995890
13	268.183120	1451234
14	280.569175	1273585
15	288.812247	1712051
16	291.149087	6775029
17	292.152424	1057009
18	305.798385	497118
19	312.879077	936532
20	312.879490	1289428

Mass Spectrum List Report

#	m/z	I
21	312.879870	587476
22	312.880239	615892
23	341.322969	1427861
24	345.020957	486894
25	354.046557	534794
26	375.454511	776974
27	398.186126	919941
28	400.199313	675630
29	400.200211	1870894
30	400.200628	3235886
31	400.201070	8314926
32	400.201827	100593712
33	400.202589	9044014
34	400.203463	2128942
35	401.198938	884674
36	401.205185	27865154
37	402.208555	3825750
38	415.025394	512578
39	417.170754	1730922
40	422.183832	1048267
41	469.316383	1405598
42	536.358390	922542
43	536.359962	758062
44	625.674256	599714
45	625.750112	2402979
46	629.442341	845023
47	630.445849	566896
48	654.650184	796031
49	750.896350	1014600
50	821.378481	3252764
51	822.381897	1499189
52	938.612030	1688852

Mass Spectrum List Report

**Analysis Info**		Acquisition Date	10/21/2025 5:31:14 PM
Analysis Name	E:\MS1 COSMIC\2025\Data_20251021\RO-7-3_pos_000001.d		
Method		Operator	Admin
Sample Name	RO-7-3	Instrument	solariX XR
Comment	RO-7-3 in MeOH C26H23N3O3 H+		

Sample Name RO-7-3 in MeOH

Exact Mass of $C_{26}H_{23}N_3O_3$ H+ = 426.181218 m/z

Mass Observed = 426.181097 m/z

Difference < 1.0 ppm

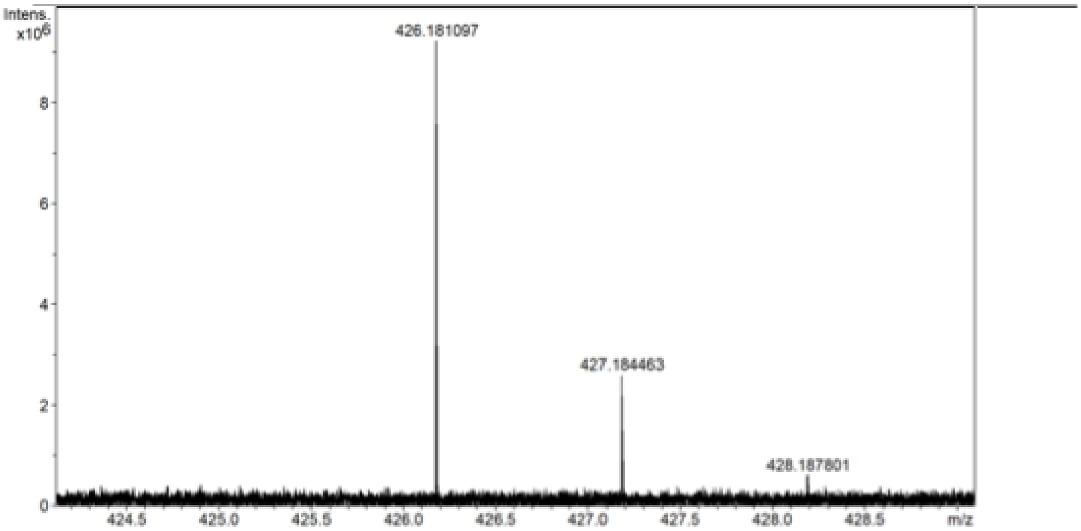

#	m/z	I
1	187.726390	455363
2	187.726787	618371
3	197.606865	1019877
4	197.607147	1171923
5	206.634589	445464
6	206.709592	458920
7	207.971002	469071
8	208.584987	1280027
9	208.585364	1241011
10	218.221088	869962
11	220.619770	478138
12	220.854884	1101334
13	234.657729	1137581
14	234.657990	1126381
15	250.301449	578570
16	250.301903	1341802
17	268.180130	1546508
18	280.569167	1325989
19	288.808982	706903
20	312.875880	1108627

Mass Spectrum List Report

#	m/z	I
21	325.827340	485231
22	341.251533	497591
23	341.318740	1110969
24	375.450557	875658
25	375.451184	547210
26	417.166635	1861420
27	426.181097	9246963
28	427.184463	2615564
29	428.187801	598693
30	469.310427	1027441
31	469.311457	1215089
32	536.353487	1483556
33	625.743580	1693437
34	625.819509	589183
35	654.649986	678331
36	750.887665	2271343
37	938.430386	574449
38	938.601797	2280959
39	979.958989	578103
